# Towards Sustainable Productivity of Greenhouse Vegetable Soils: Limiting Factors and Mitigation Strategies

**DOI:** 10.3390/plants13202885

**Published:** 2024-10-15

**Authors:** Bofang Yan, Tenghaobo Deng, Liangliang Shi

**Affiliations:** 1 Institute of Facility Agriculture, Guangdong Academy of Agricultural Sciences, Guangzhou 510640, China; yanbofang@gdaas.cn; 2 Institute of Quality Standard and Monitoring Technology for Agro-Products, Guangdong Academy of Agricultural Sciences, Guangzhou 510640, China; dengtenghb@gdaas.cn

**Keywords:** soil degradation, vegetable yield, soil organic matter, continuous cropping obstacles

## Abstract

Greenhouse vegetable production has become increasingly important in meeting the increasing global food demand. Yet, it faces severe challenges in terms of how to maintain soil productivity from a long-term perspective. This review discusses the main soil productivity limiting factors for vegetables grown in greenhouses and identifies strategies that attempt to overcome these limitations. The main processes leading to soil degradation include physical (e.g., compaction), chemical (e.g., salinization, acidification, and nutrient imbalances), and biological factors (e.g., biodiversity reduction and pathogen buildup). These processes are often favored by intensive greenhouse cultivation. Mitigation strategies involve managing soil organic matter and mineral nutrients and adopting crop rotation. Future research should focus on precisely balancing soil nutrient supply with vegetable crop demands throughout their life cycle and using targeted organic amendments to manage specific soil properties. To ensure the successful adoption of recommended strategies, socioeconomic considerations are also necessary. Future empirical research is required to adapt socioeconomic frameworks, such as Science and Technology Backyard 2.0, from cereal production systems to greenhouse vegetable production systems. Addressing these issues will enable the productivity of greenhouse vegetable soils that meet growing vegetable demand to be sustained using limited soil resources.

## 1. Introduction

Over the last decades, global vegetable demand has rapidly increased with socioeconomic development [1,2]. According to FAO (Food and Agriculture Organization of the United Nations), between 1991 and 2021, vegetable consumption per capita has almost doubled (from 76 to 147 kg), which has been paralleled by an expansion of land use for vegetable production (from 29 to 58 million hectares) [3]. These significant increases demonstrate not only the critical role of vegetable production for global food security but also the growing pressures on agricultural systems to meet this rising demand [4].

Greenhouse agriculture has become increasingly important for vegetable production, especially in the Global South after the 2000s [5]. According to the assessment using satellite data, there are 1.3 million hectares of greenhouses on the earth’s surface [5]. The largest coverage has been found in China, which accounts for 60.4% of the global greenhouse coverage and is far higher than the 5.6% in the second largest Spain. In China, about 30% of the vegetables are currently produced in greenhouses, which is aimed to increase to 40% by 2030 (data: Ministry of Agriculture and Rural Affairs of China, 2023). Greenhouses provide a controlled or semi-controlled environment for vegetable growth that prevents yield losses from extreme weather conditions (e.g., heavy rainfall and hail) and pests (e.g., *Bactrocera dorsalis*). Meanwhile, technologies such as fertigation, precise irrigation, and nutrient management are enabled in greenhouses, which minimizes leaching waste and increases resource use efficiency [6,7]. Moreover, the controlled environmental conditions in greenhouses allow for year-round vegetable production, which is not possible in open fields due to temperature and light variabilities [8]. Therefore, greenhouse vegetable production maximizes the yield and allows a stable and predictable supply to meet the vegetable demand.

A significant fraction of greenhouses are located in urban and suburban areas where the pressure on vegetable supply is especially high because of the population and the limited land resources [5]. The investments are significant in greenhouse structures as well as the associated climate control and irrigation instruments [9]. The limited land surfaces and high cost put pressure on greenhouse vegetable production to maximize yield and efficiency in resource usage to ensure economic viability [10,11]. Consequently, greenhouse vegetable production systems are characterized by high planting density, both spatially (many plants per unit area) and temporally (multiple crop cycles per year). This intensive cropping system demands substantial material inputs, including fertilizers, pesticides, and fungicides, to maintain high yields and to reduce yield losses due to pests and diseases. The intensive cropping practices in greenhouse vegetable production, while optimized for current crop growth, can contribute to stresses affecting long-term productivity.

**Figure 1:**
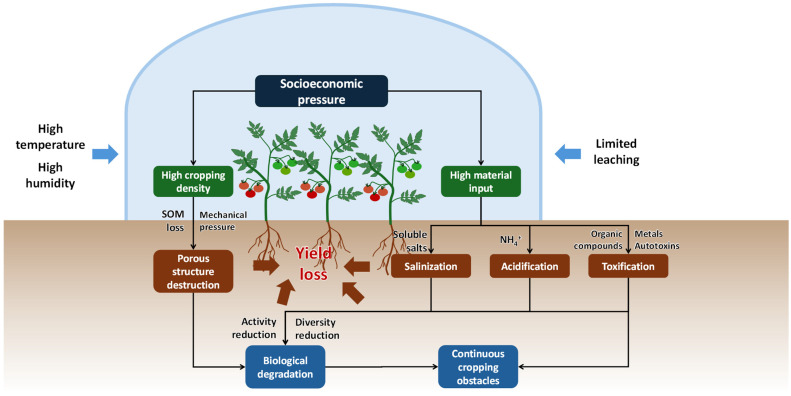
Conceptual diagram illustrating the key factors contributing to soil degradation in greenhouse vegetable production systems. Socioeconomic pressures drive high cropping density and material inputs. Physical degradation occurs through the destruction of the soil’s porous structure associated with soil organic matter (SOM) loss. Chemical degradation occurs as salinization, acidification, and toxification from accumulation of soluble salts, ammonium, metals, autotoxins, and organic compounds. These changes reduce soil microbial activity and diversity, leading to biological degradation and inducing continuous cropping obstacles. The interactions between physical, chemical, and biological factors reduce soil productivity over time and lead to yield loss of vegetable crops. The greenhouse environment, characterized by high temperature, humidity, and limited leaching, favors soil degradation processes.

Soil degradation occurs as the primary issue causing the potential decline in greenhouse vegetable productivity (Figure 1) [12,13,14]. This issue can be mainly attributed to the intensive nature of this cropping system. Previous studies have shown that overfertilization and continuous cropping can result in 30–40% yield losses in greenhouse tomato production in about 10 years or even less [15,16]. The repeated use of greenhouse soils without adequate soil management can lead to soil compaction, which reduces soil pore spaces and limits root growth and water infiltration [17]. The high input of fertilizers contributes to salt accumulation and acidification of the soil, which can suppress the uptake of soil nutrients by vegetables [18]. These changes in soil physiochemical properties can disrupt the soil microbial community, which inhibits soil nutrient cycling processes and favors the growth of microbial pathogens [19]. Moreover, the extensive use of agrochemicals can introduce toxic substances from both the chemicals themselves and their impurities (e.g., accumulated minerals, organic compounds, and heavy metal contaminants), which poses potential risks to crop and human health [18]. The soil degradation processes can even be accelerated under the unique environment in greenhouses, which is characterized by elevated temperature and humidity and protection from sunlight and rainfall. For example, the absence of rainfall limits natural leaching and retains these agrochemicals in topsoil, which accelerates soil salination, acidification, and toxification [12]. Under warm and humid conditions, soil pathogens can persist and spread rapidly [20]. The cumulative effect of these physical, chemical, and biological degradations of soils reduces the productivity of greenhouse vegetables over time. To sustain the vegetable supply, factors that contribute to soil degradation in greenhouse vegetable production systems need to be recognized and mitigated [21].

The aim of this review is to provide a synthesis of the current knowledge on the causes and processes limiting productivity in greenhouse vegetable soils and mitigation strategies for their sustainable use over crop cycles. This review does not cover the broader issues of environmental sustainability in the production of greenhouse vegetables, like carbon neutrality, soil ecosystem services, or protection of the surrounding environments, as this is well addressed in recent review papers (e.g., [12,18,22,23]). Instead, this review focuses on sustaining long-term soil productivity in greenhouse vegetable systems to meet the present and future demands.

## 2. Soil Factors Limiting Greenhouse Vegetable Productivity

### 2.1. Physical Soil Degradation

Physical degradation of soil involves the destruction of the soil’s porous structure, primarily through the breakdown of soil aggregates and the consequent compaction [24,25]. Soil aggregates are composed of soil organic and inorganic particles that are bound together. These aggregates together constitute a soil’s porous structure, which is essential for the delivery of soil water and minerals to crop roots and root expansion toward soil resources [26]. Therefore, the breakdown of soil aggregates can limit the accessibility of air, water, and nutrient resources to crops [17]. During irrigation, soil with limited water infiltration and drainage can easily be flooded, which deprives roots of oxygen and causes root rot. These factors collectively reduce plant growth and, thereby, biomass production and crop yield [27,28]. In greenhouse vegetable production systems, intensive agricultural practices often negatively impact soil aggregate stability, which makes the soil more susceptible to compaction and negatively impacts its productivity.

Multiple cropping cycles of greenhouse vegetables often mean frequent soil tillage, which is carried out between cycles and disrupts soil aggregates. Tillage involves breaking up, overturning, and mixing topsoil and subsoil, which is typically done to prepare the soil for planting and to manage crop residues (such as roots) and weeds. This practice can immediately improve the soil’s porous structure, which enhances root growth and water and fertilizer use efficiency due to increased soil aeration and resource retention [29]. However, frequent tillage repeatedly breaks down the larger aggregates into smaller particles, reducing the physical protection of macroaggregates for microaggregates and soil organic matter [24]. This impact, together with elevated soil oxygen levels, accelerates aerobic microbial decomposition processes and results in the loss of soil organic matter [25,30]. Soil organic matter, such as polysaccharides and humic substances, serves as binding agents for soil particles that form aggregates [31]. Upon frequent tillage, especially under the warm and humid conditions in greenhouses and under plastic mulches, the decomposition of this organic matter can be significant, which decreases soil aggregation and leads to physical soil degradation [32,33,34].

Many vegetable crops have fast-growing roots that can rapidly occupy the soil while exerting mechanical pressure on the surrounding soil particles with the expansion of root tips and root diameter [35]. This compressing force results in a 12–35% increase in soil bulk density in the rhizosphere (50–200 μm from the root surface) relative to that of the surrounding bulk soil [36,37]. Meanwhile, plant roots can penetrate and break large aggregates [38,39]. Nevertheless, these effects can be counterbalanced by the input of organic matter through root excretion, turnover, and fungi symbiosis, but are species-dependent [24,40]. The impact of root growth on soil aggregation can be significant in greenhouse vegetable production systems where high cropping density results in extensive root networks.

The compressing force on greenhouse soil can be derived from equipment such as tillage tractors and harvesting machinery and from the continuous trampling by workers performing planting, pruning, spraying, and harvesting tasks [41]. These machinery and human traffic are significant in the greenhouse vegetable production system, which is often labor intensive due to the high cropping density and turnover rate and the requirement for precise and continuous monitoring and management. Meanwhile, greenhouse vegetable soils are often consistently wet during crop growth to support vigorous plant growth. The controlled irrigation system, such as drip irrigation, can lead to localized soil saturation [42]. Wet soils are particularly susceptible to compaction because the presence of water fills the soil pores, reducing the cohesion and friction among soil particles and aggregates to resist dispersion when pressure is applied [17,43]. These factors combined contribute significantly to soil compaction in greenhouse vegetable production systems, negatively impacting long-term soil productivity.

Physical degradation of greenhouse vegetable soils has received relatively less attention compared to the chemical and biological factors discussed in the following sections [25]. Indeed, these factors exist interactively. For instance, the accumulation of sodium (Na) ions in soils not only causes salination but also disrupts electrostatic forces by replacing calcium (Ca) and magnesium (Mg) ions that help bind soil particles and stabilize aggregates [26]. Furthermore, soil microbial communities can affect and be influenced by soil aggregation (Figure 1). Changes in soil aggregation alter the soil microenvironment, affecting the activities of soil microbes, while microbes influence organic matter turnover that may affect soil aggregation [24]. Such interaction illustrates the complex nature of the soil degradation processes in the greenhouse vegetable production systems and requires integrated solutions toward sustainable productivity.

### 2.2. Chemical Soil Degradation

#### 2.2.1. Soil Salinization

Soil salinization refers to the accumulation of soluble salts in the soil at levels that may negatively affect plant growth and development (Figure 1). Greenhouse vegetable production often causes soil salinization due to intensive fertilization, especially with chemical fertilizers, and irrigation with poor-quality water [44,45,46]. Almost all chemical fertilizers contain salts that dissolve in soil upon application [47]. Salts are also present in organic fertilizers and other agrochemicals and can be released with their decomposition [48]. Furthermore, irrigation water may contain significant levels of salts when sourced from, for example, groundwater in arid and coastal areas [49,50]. Over time, as crops absorb only a portion of the salts input through fertilizers and irrigation water, the residual salts gradually accumulate in the soil [51,52].

Although open-field vegetable production also requires intensive fertilization and irrigation, soil salinization is less significant compared to greenhouse systems, which underlines the effect of closed greenhouse environments [53,54,55]. The absence of rainfall inside greenhouses results in limited leaching of salts out of the soil profile, which is evidenced by a reduced salt accumulation in the topsoil after opening the greenhouse to natural rainfall [54]. Moreover, evapotranspiration in greenhouses averages about 62% of that in open fields due to a significant reduction in solar radiation and ventilation [50]. This reduced evapotranspiration and precise water management (e.g., drip fertigation) further reduce water use and salt leaching in greenhouse soils. Drip fertigation, which introduces fertilizers with irrigation water, may accelerate soil salinization when crops absorb water more quickly than mineral nutrients [56]. Generally, salt accumulation in rhizosphere soil occurs during the growth stage of vegetable crops because of the high water consumption by plants with attendant depletion of water in the root zone and a lack of sufficient leaching [57]. Together, these environmental and management characteristics of greenhouse vegetable production systems often create conditions favorable to salt accumulation in the soils.

The accumulation of soluble salts in soil elevates the concentration of salts in soil solution, consequently increasing the osmotic potential. The principal ions contributing to heightened osmotic potential in arable soils include Na^+^, Ca^2+^, potassium (K^+^), Mg^2+^, nitrate (NO_3_^−^), chloride (Cl^−^), and sulfate (SO_4_^2−^) [49,55,58]. The impact of individual ions on soil osmotic potential varies. Notably, at the same concentrations, monovalent ions (e.g., Na^+^, K^+^, Cl^−^) contribute more to osmotic pressure compared to multivalent ions (e.g., Ca^2+^, Mg^2+^, SO_4_^2−^). The “Fertilizer Salt Index” serves as a valuable reference to mitigate the osmotic effects of fertilization [59]. Certain fertilizers, such as potassium chloride (KCl) and ammonium nitrate (NH_4_NO_3_), produce much higher osmotic potentials than others, such as potassium sulfate (K_2_SO_4_) and ammonium polyphosphate (APP). Under high osmotic pressure, crop roots have to consume more energy to absorb water and may even be unable to take up sufficient water in highly salinized soils [60]. Beyond osmotic stress, the toxicity induced by ions themselves can negatively influence essential physiological processes, including photosynthesis and nutrient transport. These effects will be discussed in detail in Section 2.2.4 of this review. In summary, soil salinization reduces crop dry matter production through osmotic stress and specific ion toxicity. These effects have been thoroughly reviewed e.g., by Haj-Amor et al. [58].

Salt stress resistance in vegetable crops varies significantly both between and within species and is further influenced by growth stages and environmental factors such as ventilation and humidity [61]. The review from Machado et al. provides a list of the salinity threshold among a variety of vegetable crops, where some species (e.g., broccoli (*Brassica oleracea*) and tomato (*Solanum lycopersicum*)) are more tolerant to salinity than other species (e.g., strawberry (*Fragaria × ananassa*)) [62]. Overall, most vegetable crops are poorly tolerant of salt, as yield reductions start at electrical conductivity (EC; soil saturation extracts) levels of 1–2.5 dS m^−1^. For instance, previous studies have shown that tomato yield decreased by 21% when soil EC increased from 1.1 to 5.4 dS m^−1^ [63], while pepper (*Capsicum annuum*) yield exhibited a 58% reduction when soil EC increased from 1.5 to 6.5 dS m^−1^ [64]. These examples demonstrate the importance of salinity management in greenhouse vegetable soils to ensure sustainable productivity and optimal yield.

#### 2.2.2. Soil Acidification

The high input of certain salts, namely nitrogen (N) fertilizers, can induce soil acidification. To maximize the instant vegetable yield, the application rates of N fertilizers often reach even above 4000 kg N ha^−1^ year^−1^ (e.g., in Shandong, China), while the N use efficiency is very low (<10%), resulting in excessive N accumulation in the soil [65,66]. In soils, ammonium ions (NH_4_^+^) are primarily released from urea upon hydrolyzed by urease and from ammonium-based fertilizers such as ammonium sulfate ((NH_4_)_2_SO_4_) (Figure 1). Subsequently, nitrifying bacteria (i.e., nitrite bacteria and nitrobacteria) convert NH_4_^+^ to nitrite (NO_2_^−^) and then to NO_3_^−^. Protons (H^+^) are released in this nitrification process, which lowers soil pH. Apart from chemical fertilizers, organic fertilizers also contribute significantly to soil ammonium levels, but the influence varies. For instance, clover green manure application markedly decreases soil pH, while mustard green manure has negligible effects due to differences in their ammonium contents [67]. The degree of maturation and the types of materials added during organic fertilizer composting can significantly influence the ammonium contents and acidifying potential of the fertilizer [68,69]. For example, as composting progresses, NH_4_^+^ is oxidized to NO_3_^−^, which reduces ammonium levels in the fertilizer [68]. Adding Mg^2+^ and phosphate (PO_4_^3−^) salts during composting can precipitate ammonium as struvite crystals (MgNH_4_PO_4_·6H_2_O), which preserves the ammonium within the fertilizer [69]. Lastly, crops take up NH_4_^+^ and release H^+^ to balance the charge in root cells, which can acidify the rhizosphere soil [70]. However, this acidification can be counterbalanced by NO_3_^−^ uptake, which consumes soil H^+^ and releases hydroxide ions [71]. Nevertheless, the impact on soil acidification by H^+^ release through nitrification or root exudation varies among soil types. For example, calcareous soils, which contain significant amounts of calcium carbonate (CaCO_3_), exhibit a high buffering capacity against acidification [72].

Soil acidification is a frequently reported issue in greenhouse vegetable production systems, particularly in those cropping intensively for years [18]. A study in Northern China demonstrated that excessive fertilizer application (842 kg N ha^−1^) led to the accumulation of available N in the soil and lowered soil pH from 5.7 to 4.6 over 15 years of greenhouse vegetable cultivation [73]. The same study found that after a decade of cultivation, greenhouse vegetable soils showed notably lower pH levels (5.1) compared to nearby wheat-maize fields (pH 6.5). In another study of greenhouse vegetable production, Lv et al. reported that 10 years of greenhouse cultivation resulted in pH reductions of 0.5 units in the topsoil and 0.3 units in the subsoil relative to adjacent corn fields, attributing this mainly to the accumulation and subsequent leaching of N [74]. A nationwide investigation in China revealed substantial potential acidity accumulated in greenhouse vegetable soils (approx. 230 kmol H^+^ ha^−1^ year^−1^), which is five times higher than in double-cropping cereal soils [75].

Soil pH significantly influences the chemical speciation of minerals and their interactions, such as adsorption and the formation of insoluble complexes, thereby directly affecting their availability in the soil [26]. In acidic soils (e.g., pH < 5.5), essential mineral nutrients such as phosphorus (P) and molybdenum (Mo) become less available, while the solubility of potentially toxic elements like aluminum (Al) and copper (Cu) increases [76,77]. The altered mineral availability can lead to crop nutrient deficiencies and toxicity, which suppress crop growth and productivity. Furthermore, soil acidification alters the surface charge and composition of clay minerals. These changes reduce soil cation and anion exchange capacities, which may diminish the ability of soil to retain and supply essential nutrient ions to plants [26,78]. For example, artificially lowering soil pH from 6.9 to 4.2 resulted in an 18% reduction in tomato yield [79]. Therefore, soil pH needs to be maintained at an optimal range (neutral or nearly neutral) to sustain the productivity of greenhouse vegetables.

#### 2.2.3. Soil Nutrient Imbalance

In greenhouse soils, some mineral nutrients may accumulate in excess, while others may become depleted or less available to crops [46]. Despite being essential for plant growth, high concentrations of certain mineral nutrients can interfere with the uptake and utilization of other mineral nutrients in the soil [80,81,82]. Conversely, because micronutrients are not supplied as frequently as macronutrients, and fertilizer application rates are typically calculated based on macronutrient (e.g., N) content, micronutrients can be significantly depleted from the soil [83,84,85,86].

Soil nutrient imbalance may cause antagonistic interactions between different elements [87]. For example, in plants, elevated levels of K can markedly compete with Mg and Ca for uptake pathways (i.e., root cation transporters), inhibit their uptake, and may lead to deficiencies in these elements [82,88]. Insufficient Mg supply limits chlorophyll production, which reduces photosynthetic efficiency and dry matter production [89]. Cell wall formation may be disrupted under Ca deficiency, which weakens tissue structural integrity. In vegetables like tomatoes and melons, insufficient Ca supply during fruit development can make the fruits susceptible to disorders such as blossom-end rot and fruit cracking [90,91,92]. However, at this stage, high K fertilization is often employed to enhance the sugar content in the fruit. This practice can limit Ca uptake and its subsequent translocation within the plant, increasing the risk of yield loss and fruit cracking during transportation [93,94]. For example, a greenhouse experiment with tomatoes showed a 10–15% reduction in fruit yield when the ratio of K:Ca supply increased from 0.82–0.85 to 1.00 [88]. Similarly, tomatoes grown in quartz sand substrate, supplied with nutrient solutions containing the same total concentration of macronutrients, showed a decrease in fruit yield of 15–26% when the proportion of K increased from 16% to 68% while the proportion of Ca decreased from 68% to 16% [95]. Meanwhile, depending on the cultivar, the marketable yield decreased more significantly, by 26–52%, due to an increased incidence of blossom-end rot by 17–33%. The interaction between K, Ca, and Mg in soil-plant systems needs to be carefully managed, especially in fruit vegetable production to balance the crop yield and quality.

These antagonistic effects are also observed, especially between P and certain micronutrients, such as zinc (Zn) and iron (Fe). In soils with elevated P levels, the availability of Zn and Fe is often reduced due to the formation of insoluble compounds between phosphate and metal ions. For instance, Aghili et al. reported that high P fertilization in greenhouses, as compared to open-field conditions, led to lower and deficient levels of Zn and Fe in pepper, cucumber (*Cucumis sativus*), and tomato plants [96]. Deficiencies in Zn and Fe not only reduce the nutritional value of the fruit for human consumption but also adversely affect essential physiological processes such as photosynthesis and fruit development, thereby reducing crop productivity and quality [97]. In hydroponic tomato seedlings, the highest plant yield was observed in the treatment that combined the highest Zn and the lowest P concentrations. The yield decreased by up to 80% when the log(Zn^2+^) activity decreased from −10.6 to −11.5, with more significant reductions observed as P levels increased [98]. The authors, therefore, suggest that the yield loss may also be attributed to the potentially toxic accumulation of P in the leaves. These cases about interactions between elements highlight the complexity of nutrient management in greenhouse vegetable production and indicate the importance of maintaining a balanced soil nutrient profile for sustaining long-term productivity.

#### 2.2.4. Soil Toxification

The accumulation of certain substances in greenhouse soils, while not causing salinization or acidification, may still harm crop growth and development due to their toxicity [12,18]. These toxic substances may originate from the input materials themselves, such as mineral elements from fertilizers that accumulate in the soil at toxic levels and organic compounds from pesticides. Additionally, impurities and additives in these materials can introduce pollutants, including heavy metals and organic contaminants from fertilizers and agricultural films. Moreover, some vegetable crops can excrete autotoxins, which accumulate in soil due to monocropping and harm subsequent crops of the same species. These diverse sources of soil toxicity present a significant challenge to sustainable greenhouse vegetable production, as they can significantly impact crop yield and quality (Figure 1).

The excessive use of fertilizers leads to the accumulation of mineral ions in the soil to levels that are toxic to crops. These mineral ions can be macro- and micronutrients, as well as contaminants that are not essential for higher plants. Regarding macronutrients, excessive application of ammonium-based fertilizers can lead to ammonium accumulation in soils, resulting in reduced vegetable yield due to ammonium toxicity [99]. Soil ammonium can easily transfer to ammonia and volatilize in greenhouse environments with high temperatures, insufficient ventilation, and lack of rainfall leaching. Ammonium volatilization can be especially enhanced when a nitrification inhibitor is applied to prevent the loss of ammonium and enhance the ammonium N use efficiency [100]. High concentrations of ammonia in the air (incl. soil air) can be toxic upon being absorbed through the leaves and roots [101]. In cells, ammonia reacts with water in the cytoplasm and releases ammonium and hydroxide ions, which disturb cellular pH and fundamental physiological processes such as photosynthesis. More importantly, ammonia absorbed and concentrated in the roots rapidly effluxes from root cells against the concentration gradient [102]. This influx-efflux cycle is unavailing and highly energy consuming, which causes losses of dry matter and crop yield.

Compared to macronutrients, soil trace elements are generally more risky to be toxic to plants. These elements are often transition metals. Some of them are essential for plant growth, but the sufficient levels are very close to the critical level of toxicity. For example, Cu and Zn can be toxic to crop plants at concentrations above 15 and 100 μg g^−1^ dry matter, respectively, while the concentrations of sufficiency are 5 and 30 μg g^−1^ dry matter, respectively [103]. Other trace elements, such as cadmium (Cd), are contaminants that can limit plant growth even at very low concentrations. Animal manure is the main source of these metal elements introduced into arable soils [104,105,106]. In livestock husbandry, trace elements are introduced through feed additives to prevent animal disease or reduce its severity (e.g., Cu and Zn) or through bioaccumulation by consuming contaminated feeds and pastures (e.g., Cd) [107,108]. Furthermore, wastewater irrigation, impurities in mineral fertilizers, and pesticides are also considerable sources of these potentially toxic elements in greenhouse soils, such as impure P fertilizers, which can contribute to soil Cd [109,110,111]. Isotope tracing studies have provided evidence that the buildup of these elements in topsoil is significant because only a small portion of the input can be removed from the soil through harvesting [106,112,113,114]. Greenhouse vegetable production systems are a hotspot of soil heavy metal (incl. essential micronutrients and contaminants) contamination, where higher concentrations of these elements are present in soils as compared to open-field soils due to the intensive land use and material inputs [115,116,117]. When absorbed by crops, these elements can potentially become toxic by inducing nutrient deficiency symptoms due to antagonistic effects (e.g., Zn competing with Fe, or Cd competing with Mn and Fe for transporters) [118,119]. Furthermore, elevated accumulation of these elements can trigger oxidative stress, which leads to the production of reactive oxygen species that damage cellular structure and inhibit metabolic processes [120,121]. However, although greenhouse vegetables have been shown to take up excessively these toxic elements from the soil [122], the loss of yield caused by these toxic effects is rarely reported on vegetables cultivated under field conditions. This observation suggests that the accumulation of these metals primarily impacts vegetable quality and human food safety rather than significantly affecting the productivity of greenhouse soils.

Organic contamination is a severe environmental issue in vegetable production systems because vegetables are often grown as cash crops for which farmers intensively use pesticides (e.g., insecticides and fungicides) to prevent yield losses from pests and pathogens [123]. These agrochemicals introduce organic compounds into the vegetable production system, where some of them, such as organochlorines, can remain in the soil even though their use has been prohibited for decades due to their resistance to degradation [18]. Additionally, the use of plastic films causes the contamination of phthalate acid esters, which are mixed into the plastic material in the production process as plasticizers and can readily be released and accumulated in the soil [124,125]. The controlled environment in greenhouses favors the release of these organic compounds while limiting natural degradation processes, including photodegradation, which result in a higher presence of organic contaminants in soils compared to open-field soils [126,127,128]. It has long been known that vegetable crops can take up these organic contaminants from the soil or, when volatilized, through their leaves from the air [129]. The accumulation of organic contaminants in edible parts of vegetables poses threats to human health [130], where organochlorines and leafy vegetables are of major concern [131]. While soil contamination by organic compounds raises significant ecotoxicological concerns, yield losses in vegetables due to such contamination have rarely been reported under field conditions. Therefore, similar to trace metals, the accumulation of organic contaminants in crops is not considered a major factor affecting greenhouse vegetable soil productivity.

For the economic efficiency and supply of a particular vegetable, farmers prefer to continuously grow the same species in the same field for multiple seasons without rotating with other crops [11,132]. This continuous monocropping often leads to the accumulation of autotoxins in the soil [133], which can even be accelerated in greenhouse systems by the absence of rainfall washing and limited natural degradation. Autotoxins are excreted by roots or released from degraded crop residues that can be toxic to the subsequent growing plants of the same or closely related species [134,135]. This autotoxicity is commonly observed in vegetable crops and causes growth reduction and yield losses. For example, cucumber yield was reduced by 41% after 4 years of continuous monocropping [136], and the growth of tomato seedlings was significantly limited in soils continuously used for 7 years of tomato cultivation [137]. The composition of autotoxins varies among species. In cucurbit crops, root exudates include organic acids (e.g., benzoic acid and cinnamic acid) and phenol derivatives (e.g., *p*-thiocyanatophenol) are responsible for the autotoxicity [138,139]. In tomatoes, fatty acid esters, including palmitate methyl ester and oleic acid methyl ester, respectively, affect hypocotyl and root elongation [137]. On the one hand, these autotoxins can be directly toxic to plants by inducing oxidative stress and generating reactive oxygen species, which can eventually lead to cell structure destruction [139,140]. On the other hand, crops can be more susceptible to diseases with the accumulation of autotoxins in soils. For example, cinnamic acid reduced cucumber yield by promoting the infection of roots to Fusarium wilt [141], which is similar to the eggplant (*Solanum melongena*) infection by Verticillium wilt with the presence of autotoxins cinnamic acid and vanillin [142]. A previous study on peanuts (*Arachis hypogaea*) indicates that this infection could be attributed to changes in root-colonizing microbial communities, which increased the abundance of pathogenic microbes while decreasing that of beneficial microbes [143]. In summary, continuous monocropping leads to crop exposure to species-specific autotoxins in soils, which reduce yield by limiting plant growth and increasing susceptibility to soil-borne diseases, posing significant challenges to the productivity of greenhouse vegetable soils.

### 2.3. Biological Soil Degradation

Soil is a living system where plants coexist with soil organisms, including microbes and fauna [144]. These soil organisms can be beneficial to soil fertility and crop productivity by delivering various functions, such as nutrient cycling and soil aggregate formation, while some organisms can be harmful, such as plant pathogens. Biological soil degradation is characterized by disruption of soil biological function and pathogen buildup, which is often associated with intensive land use like vegetable production [145]. As illustrated in Figure 1, the biological degradation does not occur in isolation and is often induced by the specific environmental conditions resulting from the physical and chemical degradation of soil [146,147,148]. Conversely, a reduction in soil organism population and diversity can lead to a less resilient soil ecosystem, which potentially makes the soil more susceptible to disturbance and degradation [149,150].

The functions of the soil microbes are primarily influenced by how environmental factors impact soil aggregates and the spaces between them [146]. When soils become compacted, the destruction of soil aggregates breaks the habitats of microbes and, thereby, affects the soil microbial populations [151]. The soil’s altered porous structure limits oxygen diffusion in soils. This shift towards an anaerobic environment favors the activity of anaerobic microbes such as methanogens (*Methanosarcina*) and denitrifiers, leading to greater methane and nitrous oxide production, which represent losses of organic matter and N from the soil system [146,152,153]. In addition, under the anaerobic conditions of compacted soils, the activity of iron- and sulfate-reducing bacteria (e.g., *Geobacter* and *Desulfovibrio*) can be promoted, which affects soil redox conditions and potentially leads to nutrient imbalances that affect plant growth and development [144,154]. By contrast, beneficial aerobic organisms, such as arbuscular mycorrhizal fungi (AMF) and fauna, can be depressed under low soil oxygen availability [146,155]. The impairment of symbiotic colonization of AMF on roots can negatively impact crop nutrient uptake and stress tolerance [156]. The decrease in the abundance of soil fauna, like earthworms, further compromises the physical structure of soils [24,157]. Collectively, these studies imply that physical degradation in greenhouse vegetable soils can significantly influence soil biological communities crucial for functions such as nutrient cycling and structural maintenance, which lead to decreased soil fertility and vegetable productivity.

Chemical soil degradation in greenhouse vegetable production systems also significantly influences soil biological community and function [158]. As soil salinity increases, soil fauna can be negatively affected, among which earthworms (e.g., *Eisenia fetida* and *Aporrectodea caliginosa*) are particularly sensitive to osmatic stress [159]. The decline in these organisms’ populations leads to poor soil structure, which, as discussed in Section 2.1, can adversely impact vegetable water and nutrient uptake [160]. Meanwhile, osmotic stress and the often-associated soil acidification can decrease AMF colonization (e.g., *Claroideoglomus etunicatum* and *Rhizophagus intraradices*) in vegetable roots, thereby limiting P uptake and overall plant nutrition [161,162,163,164]. Furthermore, mycorrhizal colonization has been shown to be a protective factor against soil-borne diseases in vegetable crops, such as Fusarium wilt in cucumber, and its decline can make crops more susceptible to infection [162,165]. Additionally, increased soil acidity and salinity in greenhouse vegetable soils reduce the abundance and diversity of beneficial bacteria such as *Pseudomonas* and *Bacillus* species, which play key roles in soil nutrient cycling and disease resistance [166,167,168,169]. Studies have indicated that soil acidification is a major factor contributing to crop infections caused by soil-borne pathogens [170]. For instance, in acidic soils, reduced activities of *Pseudomonas fluorescens* and *Bacillus cereus*, antagonists of the pathogen *Ralstonia solanacearum*, contribute to the outbreak of bacterial wilt disease in susceptible vegetables like tomatoes [171]. Fusarium wilt can also be induced with decreased suppression effect of *Pseudomonas* and *Bacillus* species in acidified and salinized greenhouse soils [166].

Heavy metals and persistent organic pollutants (e.g., pesticide residues) in soils can be toxic to beneficial soil organisms, which further limits the resilience and biological function of the soil ecosystem [172,173]. For example, heavy metal (e.g., Cd, Zn, and Cu) contamination can significantly reduce the diversity and colonization of soil mycorrhizal fungi [174,175], thereby affecting plant nutrient uptake and disease resistance. Multiple substances and broad-spectrum pesticides have strong impacts on soil microbial and fauna communities [176,177]. For example, fungal cells with DNA topoisomerase II can be affected by carboxylic acid fungicides, which are designed to control fungal pathogens but may act on beneficial fungi involved in soil nutrient cycling [177]. Neonicotinoid pesticides like dinotefuran and cycloxaprid can affect the survival of earthworms upon epidermal contact or food intake [178]. Meanwhile, certain pesticides exhibit increased toxicity to soil fauna either when combined with other pesticides (e.g., neonicotinoids with glyphosate) or with longer exposure periods (e.g., afidopyropen and diafenthiuron), which suggests a cumulative negative impact on soil fertility over time in greenhouse systems [176,179]. In summary, chemical soil degradation in greenhouse vegetable production systems disturbs soil organisms and their communities, which breaks crucial soil functions that facilitate plant nutrient uptake and protect against soil-borne diseases, eventually limiting vegetable yield and soil productivity.

The significant yield reduction of vegetables grown in soils with continuous monocropping (i.e., continuous cropping obstacles) can be mainly attributed to biological soil degradation, namely the development of soil-borne pathogens [133]. Long-term cultivation of the same or relative species, together with similar management practices applied over the years, simplifies the agroecosystem, which reduces soil microbial diversity [180]. For example, continuous watermelon (*Citrullus lanatus*) cropping (up to 21 years) largely affected the diversity and community structure of soil bacteria and fungi, which was significantly correlated with yield reduction [181,182]. These changes disturb the interactive balance between soil organisms and may reduce the competitive pressure on soil-borne pathogens, thereby creating conditions for their expansion [180,183]. Continuous strawberry cropping (>8 years) caused a significant shift in the composition of soil bacteria, fungi, and nematode communities toward higher fractions of phytopathogenic species [184]. Root-knot nematode disease caused by phytophagous nematodes is a commonly reported factor that causes continuous cropping obstacles for greenhouse vegetables, such as tomatoes, cucumbers, and melons (*Cucumis melo*), even when adopting crop rotation with different vegetable species [185,186,187]. Recently, Wang et al. pointed out the importance of the soil food web in the invasion of soil-borne diseases in soil continuous cropping obstacles [133], which emphasizes that understanding and managing the complex interactions within the soil ecosystem is crucial for mitigating vegetable yield losses.

## 3. Mitigating Soil Degradation toward Sustainable Greenhouse Vegetable Productivity

In agriculture, many strategies have been developed to mitigate soil degradation, such as the use of advanced materials, innovative chemical treatments, electrokinetic techniques, and phytoremediation. These strategies have been comprehensively discussed in recent reviews [22,188,189]. In the context of greenhouse vegetable production, establishing mitigation strategies requires further considerations to ensure their effectiveness and feasibility. Unlike open-field systems, greenhouse production cannot be interrupted for extended periods for soil remediation due to the pressure of investment costs and continuous demand. Moreover, although numerous materials have shown effectiveness in mitigating soil issues, only those that are readily accessible and cost-effective have the potential to be widely adopted in practice. Given these considerations, this section focuses on soil organic matter management, nutrient management, and crop rotation. These strategies have multiple benefits in mitigating soil degradation and are feasible for practical adoption, particularly when socioeconomic drivers are properly addressed. More importantly, these strategies contribute not only to addressing current issues of soil degradation but also to improving soil ecosystem resilience and long-term productivity.

### 3.1. Soil Organic Matter Management and Nutrient Management

**Figure 2:**
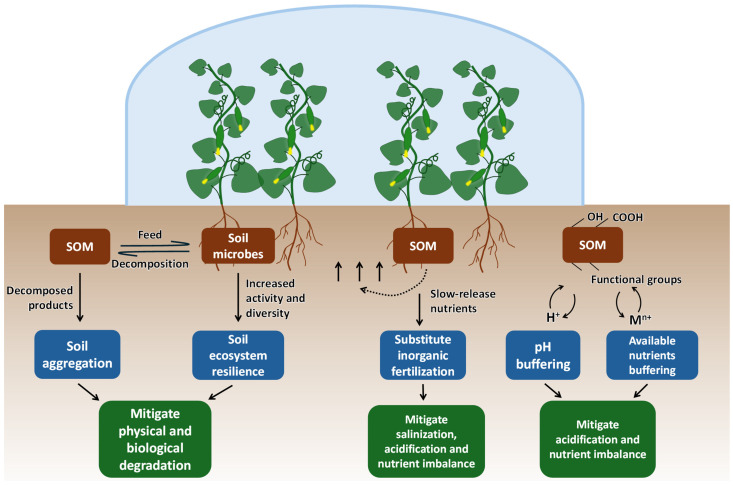
Conceptual diagram illustrating the effects of soil organic matter (SOM) on mitigating soil degradation in greenhouse vegetable production systems. SOM serves as a food source for soil microbes. The increase in soil microbial activity and diversity enhances soil aggregation and ecosystem resilience, which mitigates physical and biological degradation. SOM also provides slow-release nutrients, substituting for inorganic fertilization, which mitigates soil salinization and acidification. The functional groups in SOM contribute to pH buffering and available nutrient retention, further mitigating acidification and nutrient imbalance.

Soil organic matter management can be effective in mitigating soil degradation and maintaining stable crop production (Figure 2) [190,191]. As discussed in Section 2.1, soil organic matter is important for soil physical structure. In greenhouse vegetable soils, organic inputs such as straw and animal manure provide a primary food source for soil microbes and can significantly enhance soil microbial growth and respiration [192]. Moreover, these organic inputs can influence the soil microbial community, especially copiotrophic bacteria (e.g., *Pseudomonas* and *Bacillus* species) and saprotrophic fungi (e.g., *Trichoderma* and *Aspergillus* species) [193]. This influence can promote the decomposition of complex plant residues containing cellulose and hemicellulose, which enhances the production of binding agents such as polysaccharides and humus. These substances interact and create adhesive bonds among soil particles (e.g., clay minerals), which form soil aggregates and maintain the soil’s porous structure [24,31]. In greenhouse and open-field soils producing vegetables such as tomatoes, peppers, eggplants, and leafy greens, organic amendments have been shown to reduce soil bulk density and increase water holding capacity [194,195]. A meta analysis involving 141 studies on multiple crops including vegetables showed that manure application significantly increased soil organic matter by 18%, water-stable aggregation by 29%, and decreased soil bulk density by 4%, compared to systems using only mineral fertilizers [196]. The loose soil structure allows root growth and facilitates the delivery of water, oxygen, and nutrients toward the root surface. Therefore, maintaining the balance and level of greenhouse soil organic matter is crucial for preventing soils from physical degradation and its associated vegetable yield losses.

Soil organic matter contains various functional groups, such as carboxyl (−COOH) and phenolic hydroxyl (−OH), which can interact with ions in soil solution [197]. Depending on soil pH, these groups can accept or release H^+^, which helps buffer soil pH and, thereby, enhances soil resistance to acidification [198]. Furthermore, these functional groups create negatively charged sites, which can attract and hold mineral cations such as Ca^2+^, Mg^2+^, K^+^, and NH_4_^+^ [199]. Soil organic matter has a large surface area and can form organo-mineral complexes with clay, which further increases the density of these functional groups and enhances the soil’s ability to buffer pH and nutrient availability [26]. Previous studies have suggested that organic matter can contribute to 20–70% of total soil cation exchange capacity (CEC) [200]. This increased CEC improves nutrient retention in soils, which prevents mineral nutrients from leaching loss and makes them readily available for plant uptake. Based on a 12-year study in vegetable cultivation systems, Warman [201] reported that, compared to conventional fertilizers (i.e., inorganic N, P, and K fertilizers applied at recommended levels), compost amendment significantly increased soil pH from 5.6 to 6.4, CEC from 8.5 to 12.1 cmol kg^−1^, and Mehlich-3 extractable levels of several macro- and micronutrients by 73–147%. In addition, a 10-year study showed that partially replacing inorganic N supply with organic amendments (animal manure and straw) significantly improved soil P retention [202]. This improvement was attributed to a higher soil carbon-to-nitrogen (C/N) ratio, which enhanced microbial immobilization of P, increasing the organic P fraction while reducing the inorganic P fraction in the soil. Similarly, the long-term application of compost can nearly double the level of soil organic N [203]. This increase in the organic fraction of soil minerals slows nutrients release, which prolongs their availability to crops. Therefore, managing soil organic matter can help buffer soil pH and available nutrients, which mitigates soil acidification and nutrient imbalance.

Soil organic matter often contains multiple essential nutrients. As it decomposes over time, nutrients are slowly and continuously released for plant use [190]. Therefore, organic amendments can serve as an alternative source of mineral nutrients, partially replacing inorganic fertilizers (Figure 2). The advantages of this organic substitution have been widely reported. On the one hand, as mentioned above, the increase in soil organic matter input can improve soil structure and the ability to retain available nutrients [202,204]. On the other hand, reducing the use of inorganic fertilizers helps mitigate the soil acidification and salinization that are associated with ammonium and salt ions. A meta analysis has shown that, in greenhouse vegetable systems, substituting organic for inorganic fertilizers increased soil pH by 5% [205]. Furthermore, Zhang et al. [55] identified an inverse relationship between soil organic matter and soil salinity in greenhouse soils: areas with higher soil organic matter content tend to show lower soil salinity, and vice versa. This result confirms the mitigation effect of organic substitution on soil salinization, which can be attributed to the lower input of soluble ions and, more importantly, to the facilitated water infiltration and salt leaching thanks to the soil’s improved porous structure.

Nevertheless, under organic substitution, reducing inorganic fertilizer use may limit the supply of available mineral nutrients for the current cropping cycle. For instance, in mature compost, only about 5–15% of the N is readily available, while the majority portion is gradually released in subsequent years after application [206]. This slow release of N from organic amendments, especially for vegetable crops with a high N demand at the rapid growth stage, is a major factor limiting the yield under organic substitution [207]. To avoid yield loss, precise nutrient management is required to synchronize nutrient supply and crop demand. Management practices for N have been established for both cereal (e.g., maize) and vegetable crops, where N supply is adjusted based on modeling approaches that account for multiple cropping patterns and soil conditions [208,209]. However, compared to cereals, vegetable production relies more on meso- and micro-nutrient supply, which affects not only the yield but also resistance to diseases and product quality [210]. To date, there remains a significant knowledge gap regarding the precise nutrient requirements, particularly for micronutrients, of greenhouse vegetables throughout their growth stages, as well as the complex interactions among these elements, which is essential for optimizing yield and quality in these systems.

Soil organic matter requires dynamic management because it is not persistent and undergoes continuous decomposition [211]. This management involves regulating the decomposition of existing soil organic matter and strategically supplying new organic materials to the soil. To slow decomposition, practices such as reduced tillage and organic mulches (e.g., straw) are effective. Unlike frequent tillage and plastic mulches, these practices minimize changes in soil conditions by maintaining moderate aeration, temperature, and moisture levels [32,34,212]. While adding new organic material directly increases soil organic matter content, its impact on soil fertility and crop production varies depending on multiple factors [199]. For example, increasing the application rate of sodium-rich food waste compost introduced salinity to the soil, which resulted in decreased pepper yield compared to the lower application rate [48]. In N-fertilized soils, manure application is less efficient in increasing soil organic matter content than in N-deficient soils, since a sufficient N supply (lower soil C/N ratio) supports more active soil microbes that can more readily decompose organic inputs [213]. Compared to mature composts, organic amendments containing more microbially available C (e.g., raw manure and fresh plant residues) can be more easily decomposed, which can more rapidly release mineral nutrients and improve soil structure [214]. However, these amendments cannot provide a stable and continuous effect on soil fertility improvements over time [199,203]. Moreover, without the sustained high temperature during composting, these fresh organic amendments may contain pathogens (especially for the fresh plant residues of the same species that are affected by diseases) and weed seeds, posing a potential negative impact on crop health and yield [215]. Therefore, to achieve optimal improvements in soil fertility and vegetable yield, the application of organic amendments must be strategically managed based on both their compositional characteristics and the specific conditions of the target soil. However, in current agricultural practice, organic amendment remains, to some extent, a generalized concept, with farmers primarily relying on their source and N content for application. Further research is required to elucidate the specific interactions between organic amendment components and various soil properties, which is necessary for more precise management of soil organic matter and fertility.

### 3.2. Crop Rotation

**Figure 3:**
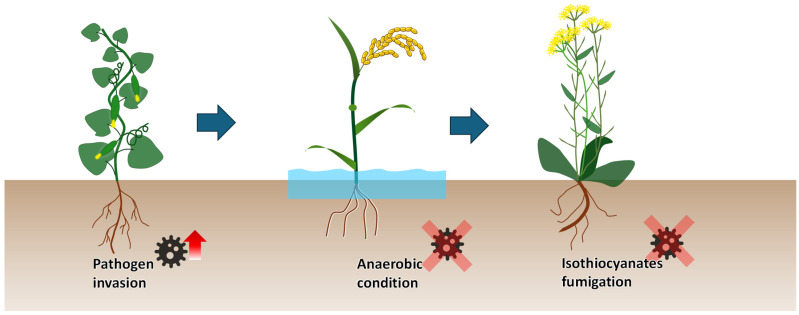
Conceptual diagram illustrating crop rotation strategies to mitigate soil-borne diseases in greenhouse vegetable production. Continuous cropping of a certain vegetable species leads to pathogen invasion. Rotation to rice creates anaerobic soil conditions, suppressing aerobic pathogens. Introduction of a Brassicaceae crop releases isothiocyanates, which act as natural soil fumigants.

As discussed in Section 2.2.4 and Section 2.3, continuous cropping obstacle is a significant problem in greenhouse vegetable production systems. This issue occurs when the same crop is grown repeatedly in the same soil, leading to yield decreases due to the accumulation of autotoxins and the development of pathogens in the soil [133]. An effective practice to overcome this issue is to adopt crop rotation, involving multiple crop species over several growing cycles (Figure 3).

Crop rotation can suppress soil-borne pathogens and enhance soil ecosystem resilience through several mechanisms. Firstly, this practice breaks the lifecycle of pathogens by introducing non-host crops. For example, rotating solanaceous crops (e.g., tomatoes, peppers, and potatoes) with non-solanaceous crops (e.g., carrots and millet) can significantly lower the incidence of soil-borne diseases like bacterial wilt by disrupting the lifecycle of *Ralstonia solanacearum* [216]. Secondly, the cultivation of certain crops in rotation can create environments hostile to pathogens. For example, the anaerobic flooding condition during rice (*Oryza sativa*) cultivation can suppress aerobic pathogens such as *Fusarium oxysporum*, which causes Fusarium wilt in tomatoes [217]. Thirdly, Brassicaceae plants like mustard, cabbage, and broccoli release sulfur-containing compounds (e.g., glucosinolates) during decomposition, which break down into isothiocyanates that can be toxic to various soil-borne pathogens, including fungi, bacteria, and nematodes [218]. Therefore, these compounds serve as a natural soil fumigation agent (i.e., biofumigation). Biofumigation provides an alternative to chemical fumigation (e.g., metham sodium) and physical disinfestation (e.g., soil steaming). These conventional methods can disrupt soil C and N cycling or create a soil biological vacuum, potentially leading to pathogen invasion [219,220]. Instead, biofumigation can influence the soil microbial community in a way that is beneficial to vegetable growth. For example, in soils under long-term pepper monoculture, rapeseed (*Brassica napus*) meal addition enhanced bacteria diversity through varying organic matter and nutrient input while reducing fungal diversity through the biocidal effect of isothiocyanates [221]. These changes could provide more biological competition against pathogens. Similar decreases in the incidence of soil-borne diseases have also been reported in diversified melon and eggplant production systems in rotation with Brassicaceae and other crops [222,223]. Furthermore, different crops with varying root systems contribute to a more diverse soil pore structure, which can improve overall soil physical quality [224]. Therefore, crop rotation is an effective practice in sustaining the productivity of greenhouse vegetable soils by preventing crops from soil-borne diseases and improving soil physicochemical properties.

### 3.3. Mitigation Strategies to Practice

Practically, the mitigation strategies have to be carefully designed and integrated with full consideration of the characteristics of greenhouse vegetable production systems and local soil and environmental conditions [12,194]. In greenhouse soils, organic matter decomposition and nutrient cycling are often accelerated by the intensive, year-round cropping in limited soil volumes, combined with controlled fertigation and often elevated temperatures [73,225]. Therefore, compared to open-field systems, more frequent and more diverse (e.g., varying decomposition rates) additions of organic materials are required to maintain the level of soil organic matter, which, as discussed above, is crucial to the soil’s porous structure, chemical buffering ability, and ecosystem resilience. Frequent organic fertilization implies a frequent nutrient input, which needs to be precisely integrated within the fertigation schedule to ensure a balanced soil nutrition status [226]. The controlled environment presents additional challenges with the potential for salinity buildup and rapid pH change, which highlights the use of organic amendments with, e.g., lower ammonium and soluble salt levels. Furthermore, the warm, humid conditions that are favorable to soil-borne pathogens further point to the need for using well-composted materials from diverse sources or even inoculating with beneficial microbes to enhance soil ability in disease resistance [227]. For example, various bacteria (e.g., *Pseudomonas fluorescens*, *Bacillus subtilis*) and fungi (e.g., *Trichoderma* species, non-pathogenic *Fusarium oxysporum*) can be used as biocontrol agents to suppress soil-borne pathogens [228]. Moreover, plant nutrition and resistance to disease can be improved by introducing arbuscular mycorrhizal fungi like *Glomus intraradices* to plant-soil systems.

For crop rotation, a well-scheduled plan should sequence species to complement each other in terms of pathogen susceptibility, nutrient use, and phenology. Such a rotation system can disrupt the life cycle of pathogens and pests, maintain a balanced soil nutrient level, and use the limited land space efficiently. Factors such as market demand and greenhouse climate control capacity need to be considered for a productive and profitable cycle each year. In southern China, for example, a widely applied rotation frame is gourd vegetables (March to July)—late rice (July to November)—Brassica vegetables (November to February), which is evident to improve soil physical properties and mitigate continuous cropping obstacles (as shown in Figure 3) [229].

To achieve an optimal and balanced nutrient supply in greenhouse vegetable soils, integrated soil management approaches are required. Foremost, regular testing of the nutritional status of soil and crops is essential to identify soil nutrient imbalances and conduct corrective measures according to soil conditions and crop requirements, e.g., adjusting fertigation schemes in real time or basing fertilization before the next crop cycle. For example, in southwestern China, Zhou et al. demonstrated that NPK fertilizer application can be reduced or even eliminated without lettuce (*Lactuca sativa*) yield loss when the concentrations of soil nitrate, Olsen-P, and exchangeable K were above the critical soil nutrient thresholds [230]. The thresholds were determined by conducting a series of experiments with varying fertilizer application rates across different soil residual nutrient levels. The nutrient supply can be taken care of steadily with minimum risk of antagonistic effects while offering a balanced supply of macro- and micronutrients using slow- or controlled-release fertilizers, ideally with a known nutrient release rate in the specific soil [231]. Additionally, between crop cycles, using cover crops or green manures can help diversify the organic inputs, which is beneficial to a resilient soil ecosystem for nutrient cycling and a balanced soil nutrient supply. In case of severe nutrient imbalances occur on crops, foliar fertilization provides a rapid correction before the soil mitigation measures are carried out [232].

In sum, in practical implementation, these mitigation measures should be integrated and dynamically adjusted according to regular soil and crop tests and the changing greenhouse environments. An in-depth understanding of the interactions among soils, crops, and amendments is essential to effectively and simultaneously address multiple soil degradation issues and create a resilient greenhouse system that is sustainable for long-term vegetable production.

## 4. Concluding Remarks

According to Giampietro [11], socioeconomic pressures, including demographic growth, economic demands for higher labor productivity, and market forces favoring specialization, are major drivers that push farmers toward high-input and high-yielding cropping intensification. These practices favor short-term rather than long-term soil productivity, potentially leading to soil nutrient depletion, structural disruption, and loss of biodiversity in the agroecosystem. According to the discussion in the preceding sections, it is evident that modifying fertilization practices (including organic substitution and precise nutrient supply) and adjusting cropping patterns (such as rotation) are basic approaches to mitigate physical, chemical, and biological soil degradation and sustaining the productivity of greenhouse vegetable soils. For more precise management of greenhouse vegetable soils, a better understanding of nutrient requirements for vegetable crops throughout their growth stages is required, particularly regarding plant meso- and micronutrients. Additionally, it remains to improve the model of the complex interactions between organic amendment components and the soil properties to be managed.

The recommended mitigation strategies will only be feasible if there is an understanding of their prospective socioeconomic challenges and the incorporation of relevant socioeconomic factors in their adoption. Recently, An et al. introduced a framework for identifying and overcoming barriers to farmers’ adoption of recommended practices, informed by decades of experience from “the Science and Technology Backyard (STB)” platform [233]. This framework, called STB 2.0, emphasizes the importance of farmer-centered dialogue and multistakeholder involvement. Equal and open dialogue not only bridges the knowledge gap between scientists and farmers but also helps adapt recommended practices to farmers’ specific experiences, concerns, and local conditions. Furthermore, multistakeholder collaboration can better align government support with the interests of farmers and suppliers of agricultural materials, thereby increasing the feasibility of recommended practices by enabling benefit-sharing and distributing risks related to crop failure and resource supply instability.

While this STB 2.0 framework has proven effective for wheat and maize production, its application to greenhouse vegetables requires further adjustment and development. Several challenges may arise in the context of greenhouse vegetable production, including higher market fluctuations in product demand and price, as well as the diverse crop species requiring varying and more frequent adjusted management, which makes it difficult to develop standardized recommendation practices. Moreover, unlike staple cereals that focus primarily on yield, vegetable crops often prioritize quality attributes. This emphasis on quality necessitates infrastructure investments and intensive labor, representing higher capital input and potentially increasing farmers’ perceived financial risk in adopting novel practices. To address these challenges, potential adaptations for greenhouse vegetable production systems should include focusing on quality management and emphasizing market-oriented collaborations that involve stakeholders from the entire value chain (including retailers and consumers) to overcome market uncertainties. Furthermore, creating crop-specific sub-networks within the STB 2.0 framework could help to recommend customized practices and provide specialized support. Such support and collaboration should be long-standing and maintained throughout the entire process of greenhouse vegetable production, which is essential to provide farmers with the confidence to invest in long-term practices that sustain soil productivity.

## References

[1] Mello Rodrigues V., Bray J., Fernandes A.C., Luci Bernardo G., Hartwell H., Secchi Martinelli S., Lazzarin Uggioni P., Barletto Cavalli S., Proença R.P.D.C. (2019). Vegetable Consumption and Factors Associated with Increased Intake among College Students: A Scoping Review of the Last 10 Years. Nutrients.

[2] Affret A., Severi G., Dow C., Mancini F.R., Rey G., Delpierre C., Clavel-Chapelon F., Boutron-Ruault M.-C., Fagherazzi G. (2018). Socio-economic factors associated with an increase in fruit and vegetable consumption: A 12-year study in women from the E3N-EPIC study. Public Health Nutr..

[3] FAO (2023). Food Balances: Food Balances (2010–).

[4] FAO, IFAD, UNICEF, WFP, WHO (2023). The State of Food Security and Nutrition in the World 2023. Urbanization, Agrifood Systems Transformation and Healthy Diets Across the Rural–Urban Continuum.

[5] Tong X., Zhang X., Fensholt R., Jensen P.R.D., Li S., Larsen M.N., Reiner F., Tian F., Brandt M. (2024). Global area boom for greenhouse cultivation revealed by satellite mapping. Nat. Food.

[6] Yuan Y., Zhang X. (2021). Comparison of agrochemicals allocation efficiency between greenhouse and open-field vegetables in China. Sci. Rep..

[7] Nikolaou G., Neocleous D., Christou A., Polycarpou P., Kitta E., Katsoulas N. (2021). Energy and Water Related Parameters in Tomato and Cucumber Greenhouse Crops in Semiarid Mediterranean Regions. A Review, Part II: Irrigation and Fertigation. Horticulturae.

[8] Nemali K. (2022). History of Controlled Environment Horticulture: Greenhouses. HortScience.

[9] Kuswardhani N., Soni P., Shivakoti G.P. (2013). Comparative energy input–output and financial analyses of greenhouse and open field vegetables production in West Java, Indonesia. Energy.

[10] Hollingsworth J.A., Ravishankar E., O’Connor B., Johnson J.X., DeCarolis J.F. (2020). Environmental and economic impacts of solar-powered integrated greenhouses. J. Ind. Ecol..

[11] Giampietro M. (1997). Socioeconomic constraints to farming with biodiversity. Agric. Ecosyst. Environ..

[12] Hu W., Zhang Y., Huang B., Teng Y. (2017). Soil environmental quality in greenhouse vegetable production systems in eastern China: Current status and management strategies. Chemosphere.

[13] Liu X., Li Y., Ren X., Chen B., Zhang Y., Shen C., Wang F., Wu D. (2020). Long-Term Greenhouse Cucumber Production Alters Soil Bacterial Community Structure. J. Soil Sci. Plant Nutr..

[14] Qasim W., Xia L., Lin S., Wan L., Zhao Y., Butterbach-Bahl K. (2021). Global greenhouse vegetable production systems are hotspots of soil N_2_O emissions and nitrogen leaching: A meta-analysis. Environ. Pollut..

[15] Song Q., Fu H., Shi Q., Shan X., Wang Z., Sun Z., Li T. (2022). Overfertilization reduces tomato yield under long-term continuous cropping system via regulation of soil microbial community composition. Front. Microbiol..

[16] Fu H., Zhang G., Zhang F., Sun Z., Geng G., Li T. (2017). Effects of Continuous Tomato Monoculture on Soil Microbial Properties and Enzyme Activities in a Solar Greenhouse. Sustainability.

[17] Hamza M.A., Anderson W.K. (2005). Soil compaction in cropping systems: A review of the nature, causes and possible solutions. Soil Tillage Res..

[18] Kianpoor Kalkhajeh Y., Huang B., Hu W., Ma C., Gao H., Thompson M.L., Bruun Hansen H.C. (2021). Environmental soil quality and vegetable safety under current greenhouse vegetable production management in China. Agric. Ecosyst. Environ..

[19] Coban O., De Deyn G.B., van der Ploeg M. (2022). Soil microbiota as game-changers in restoration of degraded lands. Science.

[20] Xin X.-F., Nomura K., Aung K., Velásquez A.C., Yao J., Boutrot F., Chang J.H., Zipfel C., He S.Y. (2016). Bacteria establish an aqueous living space in plants crucial for virulence. Nature.

[21] Lamichhane J.R. (2024). Greenhouse cultivation for more sustainable food systems. Nat. Food.

[22] Hou D., Bolan N.S., Tsang D.C., Kirkham M.B., O’connor D. (2020). Sustainable soil use and management: An interdisciplinary and systematic approach. Sci. Total Environ..

[23] Mazibuko D.M., Gono H., Maskey S., Okazawa H., Fiwa L., Kikuno H., Sato T. (2023). The Sustainable Niche for Vegetable Production within the Contentious Sustainable Agriculture Discourse: Barriers, Opportunities and Future Approaches. Sustainability.

[24] Six J., Bossuyt H., Degryze S., Denef K. (2004). A history of research on the link between (micro)aggregates, soil biota, and soil organic matter dynamics. Soil Tillage Res..

[25] Wang M., Xu S., Yang J., Xu L., Yu Q., Xie X., Shi X., Zhao Y. (2021). The effect of organic and conventional management practices on soil macropore structure in greenhouse vegetable production. Eur. J. Soil Sci..

[26] Weil R.R., Brady N.C. (2016). The Nature and Properties of Soils.

[27] Hefner M., Labouriau R., Nørremark M., Kristensen H.L. (2019). Controlled traffic farming increased crop yield, root growth, and nitrogen supply at two organic vegetable farms. Soil Tillage Res..

[28] Johansen T.J., Thomsen M.G., Løes A.-K., Riley H. (2015). Root development in potato and carrot crops—Influences of soil compaction. Acta Agric. Scand. Sect. B Soil Plant Sci..

[29] Grasso R., Peña-Fleitas M.T., Gallardo M., Thompson R.B., Padilla F.M. (2021). Tillage effects on soil properties, crop responses and root density of sweet pepper (*Capsicum annuum*). Span. J. Agric. Res..

[30] Man M., Wagner-Riddle C., Dunfield K.E., Deen B., Simpson M.J. (2021). Long-term crop rotation and different tillage practices alter soil organic matter composition and degradation. Soil Tillage Res..

[31] Bongiovanni M.D., Lobartini J.C. (2006). Particulate organic matter, carbohydrate, humic acid contents in soil macro- and microaggregates as affected by cultivation. Geoderma.

[32] Redmile-Gordon M., Gregory A.S., White R.P., Watts C.W. (2020). Soil organic carbon, extracellular polymeric substances (EPS), and soil structural stability as affected by previous and current land-use. Geoderma.

[33] Chen Q., Zhou Z., Cai S., Lv M., Yang Y., Luo Y., Jiang H., Liu R., Cao T., Yao B. (2024). Spatial-temporal variation of soil organic matter decomposition potential in China. Soil Tillage Res..

[34] Steinmetz Z., Wollmann C., Schaefer M., Buchmann C., David J., Tröger J., Muñoz K., Frör O., Schaumann G.E. (2016). Plastic mulching in agriculture. Trading short-term agronomic benefits for long-term soil degradation?. Sci. Total Environ..

[35] Zapata-Sierra A.J., Moreno-Pérez M.F., Reyes-Requena R., Manzano-Agugliaro F. (2021). Root distribution with the use of drip irrigation on layered soils at greenhouses crops. Sci. Total Environ..

[36] Young I. (1998). Biophysical interactions at the root–soil interface: A review. J. Agric. Sci..

[37] Dorioz J.M., Robert M., Chenu C. (1993). The role of roots, fungi and bacteria on clay particle organization. An experimental approach. Geoderma.

[38] Materechera S., Kirby J., Alston A., Dexter A. (1994). Modification of soil aggregation by watering regime and roots growing through beds of large aggregates. Plant Soil.

[39] Kolb E., Quiros M., Meijer G.J., Bogeat-Triboulot M.B., Carminati A., Andò E., Sibille L., Anselmucci F., Jensen K., Forterre Y. (2022). Root–Soil Interaction. Soft Matter in Plants: From Biophysics to Biomimetics.

[40] Rillig M.C., Aguilar-Trigueros C.A., Bergmann J., Verbruggen E., Veresoglou S.D., Lehmann A. (2015). Plant root and mycorrhizal fungal traits for understanding soil aggregation. New Phytol..

[41] McPhee J.E., Aird P.L., Hardie M.A., Corkrey S.R. (2015). The effect of controlled traffic on soil physical properties and tillage requirements for vegetable production. Soil Tillage Res..

[42] Reyes-Cabrera J., Zotarelli L., Dukes M.D., Rowland D.L., Sargent S.A. (2016). Soil moisture distribution under drip irrigation and seepage for potato production. Agric. Water Manag..

[43] Batey T. (2009). Soil compaction and soil management—A review. Soil Use Manag..

[44] Wu R., Sun H., Xue J., Yan D., Liu Y., Gui D., Wang X., Yang J. (2020). Acceleration of soil salinity accumulation and soil degradation due to greenhouse cultivation: A survey of farmers’ practices in China. Environ. Monit. Assess..

[45] Sun K., Zhang J., Zhang W., Zhou W., Wang J. (2023). Characteristics of Soil Salinity in Representative Plastic Shed Vegetable Production Areas in Shandong Province, China. Eurasian Soil Sci..

[46] Bai X., Gao J., Wang S., Cai H., Chen Z., Zhou J. (2020). Excessive nutrient balance surpluses in newly built solar greenhouses over five years leads to high nutrient accumulations in soil. Agric. Ecosyst. Environ..

[47] Han J., Shi J., Zeng L., Xu J., Wu L. (2015). Effects of nitrogen fertilization on the acidity and salinity of greenhouse soils. Environ. Sci. Pollut. Res..

[48] Lee C.H., Park S.J., Hwang H.Y., Kim M.S., Jung H.i., Luyima D., Hong S.Y., Oh T.K., Kim S.H. (2019). Effects of food waste compost on the shift of microbial community in water saturated and unsaturated soil condition. Appl. Biol. Chem..

[49] Yasuor H., Yermiyahu U., Ben-Gal A. (2020). Consequences of irrigation and fertigation of vegetable crops with variable quality water: Israel as a case study. Agric. Water Manag..

[50] Phogat V., Mallants D., Cox J.W., Šimůnek J., Oliver D.P., Awad J. (2020). Management of soil salinity associated with irrigation of protected crops. Agric. Water Manag..

[51] Yan Z., Liu P., Li Y., Ma L., Alva A., Dou Z., Chen Q., Zhang F. (2013). Phosphorus in China’s Intensive Vegetable Production Systems: Overfertilization, Soil Enrichment, and Environmental Implications. J. Environ. Qual..

[52] Douxchamps S., Frossard E., Bernasconi S.M., van der Hoek R., Schmidt A., Rao I.M., Oberson A. (2011). Nitrogen recoveries from organic amendments in crop and soil assessed by isotope techniques under tropical field conditions. Plant Soil.

[53] Huang S.-w., Gao W., Tang J.-w., Li C.-h. (2016). Total salt content and ion composition in tillage layer of soils in the main vegetable production regions of China. J. Plant Nutr. Fertil..

[54] Sun H., Wei C., Xu W., Yang J., Wang X., Qiu Y. (2019). Characteristics of salt contents in soils under greenhouse conditions in China. Environ. Sci. Pollut. Res..

[55] Zhang Z., Sun D., Tang Y., Zhu R., Li X., Gruda N., Dong J., Duan Z. (2021). Plastic shed soil salinity in China: Current status and next steps. J. Clean. Prod..

[56] Yan F., Zhang F., Fan X., Fan J., Wang Y., Zou H., Wang H., Li G. (2021). Determining irrigation amount and fertilization rate to simultaneously optimize grain yield, grain nitrogen accumulation and economic benefit of drip-fertigated spring maize in northwest China. Agric. Water Manag..

[57] Zhang Y., Li X., Šimůnek J., Shi H., Chen N., Hu Q., Tian T. (2021). Evaluating soil salt dynamics in a field drip-irrigated with brackish water and leached with freshwater during different crop growth stages. Agric. Water Manag..

[58] Haj-Amor Z., Araya T., Kim D.-G., Bouri S., Lee J., Ghiloufi W., Yang Y., Kang H., Jhariya M.K., Banerjee A. (2022). Soil salinity and its associated effects on soil microorganisms, greenhouse gas emissions, crop yield, biodiversity and desertification: A review. Sci. Total Environ..

[59] Killorn R., Voss R.D. (1986). Iowa State University Cooperative Extension. Salt Index of Fertilizers.

[60] Taiz L., Møller I.M., Murphy A.S., Zeiger E. (2023). Plant Physiology and Development.

[61] Shannon M.C., Grieve C.M. (1998). Tolerance of vegetable crops to salinity. Sci. Hortic..

[62] Machado R.M.A., Serralheiro R.P. (2017). Soil Salinity: Effect on Vegetable Crop Growth. Management Practices to Prevent and Mitigate Soil Salinization. Horticulturae.

[63] Boari F., Cantore V., Di Venere D., Sergio L., Candido V., Schiattone M.I. (2019). Pyraclostrobin can mitigate salinity stress in tomato crop. Agric. Water Manag..

[64] Huez-López M.A., Ulery A.L., Samani Z., Picchioni G., Flynn R. (2011). Response of chile pepper (*Capsicum annuum* L.) to salt stress and organic and inorganic nitrogen sources: I. Growth and yield. Trop. Subtrop. Agroecosyst..

[65] Ju X.T., Kou C.L., Christie P., Dou Z., Zhang F. (2007). Changes in the soil environment from excessive application of fertilizers and manures to two contrasting intensive cropping systems on the North China Plain. Environ. Pollut..

[66] Zhu J., Li X., Christie P., Li J. (2005). Environmental implications of low nitrogen use efficiency in excessively fertilized hot pepper (*Capsicum frutescens* L.) cropping systems. Agric. Ecosyst. Environ..

[67] Grüter R., Meister A., Schulin R., Tandy S. (2017). Green manure effects on zinc and cadmium accumulation in wheat grains (*Triticum aestivum* L.) on high and low zinc soils. Plant Soil.

[68] Azim K., Soudi B., Boukhari S., Perissol C., Roussos S., Thami Alami I. (2018). Composting parameters and compost quality: A literature review. Org. Agric..

[69] Sánchez Ó.J., Ospina D.A., Montoya S. (2017). Compost supplementation with nutrients and microorganisms in composting process. Waste Manag..

[70] Song J., Yang J., Jeong B.R. (2022). Decreased Solution pH and Increased K^+^ Uptake Are Related to Ammonium Tolerance in Hydroponically Cultured Plants. Horticulturae.

[71] Ye J.Y., Tian W.H., Zhou M., Zhu Q.Y., Du W.X., Zhu Y.X., Liu X.X., Lin X.Y., Zheng S.J., Jin C.W. (2021). STOP1 activates NRT1. 1-mediated nitrate uptake to create a favorable rhizospheric pH for plant adaptation to acidity. Plant Cell.

[72] Wang Z., Jia M., Li Z., Liu H., Christie P., Wu L. (2020). Acid buffering capacity of four contrasting metal-contaminated calcareous soil types: Changes in soil metals and relevance to phytoextraction. Chemosphere.

[73] Li J., Wan X., Liu X., Chen Y., Slaughter L.C., Weindorf D.C., Dong Y. (2019). Changes in soil physical and chemical characteristics in intensively cultivated greenhouse vegetable fields in North China. Soil Tillage Res..

[74] Lv H., Zhao Y., Wang Y., Wan L., Wang J., Butterbach-Bahl K., Lin S. (2020). Conventional flooding irrigation and over fertilization drives soil pH decrease not only in the top- but also in subsoil layers in solar greenhouse vegetable production systems. Geoderma.

[75] Guo J.H., Liu X.J., Zhang Y., Shen J.L., Han W.X., Zhang W.F., Christie P., Goulding K.W.T., Vitousek P.M., Zhang F.S. (2010). Significant Acidification in Major Chinese Croplands. Science.

[76] Coskun D., White P.J., Rengel Z., Cakmak I., White P.J. (2023). Chapter 2—Ion-uptake mechanisms of individual cells and roots: Short-distance transport. This chapter is a revision of the third edition chapter by P.J. White, pp. 7–47. Marschner’s Mineral Nutrition of Plants.

[77] White P.J., Broadley M.R. (2009). Biofortification of crops with seven mineral elements often lacking in human diets—Iron, zinc, copper, calcium, magnesium, selenium and iodine. New Phytol..

[78] Yin N., Geng N., Wang T., Wang H., Pan H., Yang Q., Lou Y., Zhuge Y. (2022). Effect of acidification on clay minerals and surface properties of brown soil. Sustainability.

[79] Zhang X., Shan X., Fu H., Sun Z. (2022). Effects of artificially-simulated acidification on potential soil nitrification activity and ammonia oxidizing microbial communities in greenhouse conditions. PeerJ.

[80] Fan Y.n., Zhang Y., Hess F., Huang B., Chen Z. (2020). Nutrient balance and soil changes in plastic greenhouse vegetable production. Nutr. Cycl. Agroecosyst..

[81] Wan L., Lv H., Qasim W., Xia L., Yao Z., Hu J., Zhao Y., Ding X., Zheng X., Li G. (2022). Heavy metal and nutrient concentrations in top- and sub-soils of greenhouses and arable fields in East China—Effects of cultivation years, management, and shelter. Environ. Pollut..

[82] Xie K., Cakmak I., Wang S., Zhang F., Guo S. (2021). Synergistic and antagonistic interactions between potassium and magnesium in higher plants. Crop J..

[83] Mondal A., Rai A., Wali P., Kumar M. (2015). Available micronutrient status and their relationship with soil properties of vegetable growing area of Jammu district. Progress. Hortic..

[84] Graham R.D., Alloway B.J. (2008). Micronutrient Deficiencies in Crops and Their Global Significance. Micronutrient Deficiencies in Global Crop Production.

[85] Shiwakoti S., Zheljazkov V.D., Gollany H.T., Kleber M., Xing B. (2019). Micronutrients decline under long-term tillage and nitrogen fertilization. Sci. Rep..

[86] Asrade D.A., Kulhánek M., Černý J., Sedlář O., Balík J. (2022). Effects of long-term mineral fertilization on silage maize monoculture yield, phosphorus uptake and its dynamic in soil. Field Crops Res..

[87] Rietra R.P., Heinen M., Dimkpa C.O., Bindraban P.S. (2017). Effects of nutrient antagonism and synergism on yield and fertilizer use efficiency. Commun. Soil Sci. Plant Anal..

[88] Hernández-Pérez O.I., Valdez-Aguilar L.A., Alia-Tejacal I., Cartmill A.D., Cartmill D.L. (2020). Tomato fruit yield, quality, and nutrient status in response to potassium: Calcium balance and electrical conductivity in the nutrient solution. J. Soil Sci. Plant Nutr..

[89] Meng X., Bai S., Wang S., Pan Y., Chen K., Xie K., Wang M., Guo S. (2023). The sensitivity of photosynthesis to magnesium deficiency differs between rice (*Oryza sativa* L.) and cucumber (*Cucumis sativus* L.). Front. Plant Sci..

[90] Lopez-Zaplana A., Bárzana G., Ding L., Chaumont F., Carvajal M. (2022). Aquaporins involvement in the regulation of melon (*Cucumis melo* L.) fruit cracking under different nutrient (Ca, B and Zn) treatments. Environ. Exp. Bot..

[91] Saure M. (2001). Blossom-end rot of tomato (*Lycopersicon esculentum* Mill.)—A calcium-or a stress-related disorder?. Sci. Hortic..

[92] Taylor M.D., Locascio S.J. (2004). Blossom-end rot: A calcium deficiency. J. Plant Nutr..

[93] Liu J., Hu T., Feng P., Wang L., Yang S. (2019). Tomato yield and water use efficiency change with various soil moisture and potassium levels during different growth stages. PLoS ONE.

[94] Kuzin A., Solovchenko A. (2021). Essential Role of Potassium in Apple and Its Implications for Management of Orchard Fertilization. Plants.

[95] Fanasca S., Colla G., Maiani G., Venneria E., Rouphael Y., Azzini E., Saccardo F. (2006). Changes in antioxidant content of tomato fruits in response to cultivar and nutrient solution composition. J. Agric. Food Chem..

[96] Aghili F., Khoshgoftarmanesh A.H., Afyuni M., Mobli M. (2012). Mineral and Ascorbic Acid Concentrations of Greenhouse- and Field-Grown Vegetables: Implications for Human Health. Int. J. Veg. Sci..

[97] Hamzah Saleem M., Usman K., Rizwan M., Al Jabri H., Alsafran M. (2022). Functions and strategies for enhancing zinc availability in plants for sustainable agriculture. Front. Plant Sci..

[98] Parker D.R., Aguilera J.J., Thomason D.N. (1992). Zinc-phosphorus interactions in two cultivars of tomato (*Lycopersicon esculentum* L.) grown in chelator-buffered nutrient solutions. Plant Soil.

[99] Wang Y., Zhang X., Liu H., Sun G., Song S., Chen R. (2022). High NH_4_^+^/NO_3_^−^ Ratio Inhibits the Growth and Nitrogen Uptake of Chinese Kale at the Late Growth Stage by Ammonia Toxicity. Horticulturae.

[100] Min J., Sun H., Kronzucker H.J., Wang Y., Shi W. (2021). Comprehensive assessment of the effects of nitrification inhibitor application on reactive nitrogen loss in intensive vegetable production systems. Agric. Ecosyst. Environ..

[101] Coskun D., Britto D.T., Li M., Becker A., Kronzucker H.J. (2013). Rapid ammonia gas transport accounts for futile transmembrane cycling under NH_3_/NH_4_^+^ toxicity in plant roots. Plant Physiol..

[102] Britto D.T., Siddiqi M.Y., Glass A.D.M., Kronzucker H.J. (2001). Futile transmembrane NH_4_^+^ cycling: A cellular hypothesis to explain ammonium toxicity in plants. Proc. Natl. Acad. Sci. USA.

[103] White P.J., Brown P.H. (2010). Plant nutrition for sustainable development and global health. Ann. Bot..

[104] Luo L., Ma Y., Zhang S., Wei D., Zhu Y.G. (2009). An inventory of trace element inputs to agricultural soils in China. J. Environ. Manag..

[105] Zhao F.J., Ma Y., Zhu Y.G., Tang Z., McGrath S.P. (2015). Soil contamination in China: Current status and mitigation strategies. Environ. Sci. Technol..

[106] Imseng M., Wiggenhauser M., Muller M., Keller A., Frossard E., Wilcke W., Bigalke M. (2019). The fate of Zn in agricultural soils: A stable isotope approach to anthropogenic impact, soil formation, and soil-plant cycling. Environ. Sci. Technol..

[107] Brugger D., Windisch W.M. (2015). Environmental responsibilities of livestock feeding using trace mineral supplements. Anim. Nutr..

[108] Loganathan P., Hedley M.J., Grace N.D., Whitacre D.M. (2008). Pasture Soils Contaminated with Fertilizer-Derived Cadmium and Fluorine: Livestock Effects. Reviews of Environmental Contamination and Toxicology.

[109] Pinot F., Kreps S.E., Bachelet M., Hainaut P., Bakonyi M., Polla B.S. (2000). Cadmium in the environment: Sources, mechanisms of biotoxicity, and biomarkers. Rev. Environ. Health.

[110] Wang Z., Li J., Li Y. (2017). Using Reclaimed Water for Agricultural and Landscape Irrigation in China: A Review. Irrig. Drain..

[111] Chen Z., Imran M., Jing G., Wang W., Huang B., Li Y., Zhang Y., Yang Y., Lu Q., Zhang Z. (2023). Toxic elements pollution risk as affected by various input sources in soils of greenhouses, kiwifruit orchards, cereal fields, and forest/grassland. Environ. Pollut..

[112] Ostermann A., He Y., Siemens J., Welp G., Heuser A., Wombacher F., Munker C., Xue Q., Lin X., Amelung W. (2015). Tracing copper derived from pig manure in calcareous soils and soil leachates by ^65^Cu labeling. Environ. Sci. Technol..

[113] Wiggenhauser M., Bigalke M., Imseng M., Keller A., Rehkämper M., Wilcke W., Frossard E. (2019). Using isotopes to trace freshly applied cadmium through mineral phosphorus fertilization in soil-fertilizer-plant systems. Sci. Total Environ..

[114] Yan B.-F., Dürr-Auster T., Frossard E., Wiggenhauser M. (2021). The Use of Stable Zinc Isotope Soil Labeling to Assess the Contribution of Complex Organic Fertilizers to the Zinc Nutrition of Ryegrass. Front. Plant Sci..

[115] Zhang X., Song X., Zhang H., Li Y., Hou Y., Zhao X. (2023). Source apportionment and risk assessment of heavy metals in typical greenhouse vegetable soils in Shenyang, China. Environ. Monit. Assess..

[116] Su C., Wang J., Chen Z., Meng J., Yin G., Zhou Y., Wang T. (2023). Sources and health risks of heavy metals in soils and vegetables from intensive human intervention areas in South China. Sci. Total Environ..

[117] Liu J., Wang Y., Liu X., Xu J. (2021). Occurrence and health risks of heavy metals in plastic-shed soils and vegetables across China. Agric. Ecosyst. Environ..

[118] Assuncao A.G.L., Cakmak I., Clemens S., Gonzalez-Guerrero M., Nawrocki A., Thomine S. (2022). Micronutrient homeostasis in plants for more sustainable agriculture and healthier human nutrition. J. Exp. Bot..

[119] Stanton C., Sanders D., Kramer U., Podar D. (2022). Zinc in plants: Integrating homeostasis and biofortification. Mol. Plant.

[120] Kumar V., Pandita S., Singh Sidhu G.P., Sharma A., Khanna K., Kaur P., Bali A.S., Setia R. (2021). Copper bioavailability, uptake, toxicity and tolerance in plants: A comprehensive review. Chemosphere.

[121] Verbruggen N., Hermans C., Schat H. (2009). Mechanisms to cope with arsenic or cadmium excess in plants. Curr. Opin. Plant Biol..

[122] Li F.-L., Shi W., Jin Z.-F., Wu H.-M., Sheng G.D. (2017). Excessive uptake of heavy metals by greenhouse vegetables. J. Geochem. Explor..

[123] Dinham B. (2003). Growing vegetables in developing countries for local urban populations and export markets: Problems confronting small-scale producers. Pest Manag. Sci..

[124] Wang J., Chen G., Christie P., Zhang M., Luo Y., Teng Y. (2015). Occurrence and risk assessment of phthalate esters (PAEs) in vegetables and soils of suburban plastic film greenhouses. Sci. Total Environ..

[125] Cui J., Bai R., Ding W., Liu Q., Liu Q., He W., Yan C., Li Z. (2024). Potential agricultural contamination and environmental risk of phthalate acid esters arrived from plastic film mulching. J. Environ. Chem. Eng..

[126] Lü H., Mo C.-H., Zhao H.-M., Xiang L., Katsoyiannis A., Li Y.-W., Cai Q.-Y., Wong M.-H. (2018). Soil contamination and sources of phthalates and its health risk in China: A review. Environ. Res..

[127] Li Z., Sun J., Zhu L. (2021). Organophosphorus pesticides in greenhouse and open-field soils across China: Distribution characteristic, polluted pathway and health risk. Sci. Total Environ..

[128] Dou R., Sun J., Deng F., Wang P., Zhou H., Wei Z., Chen M., He Z., Lai M., Ye T. (2020). Contamination of pyrethroids and atrazine in greenhouse and open-field agricultural soils in China. Sci. Total Environ..

[129] Trapp S., Legind C.N., Swartjes F.A. (2011). Uptake of Organic Contaminants from Soil into Vegetables and Fruits. Dealing with Contaminated Sites: From Theory towards Practical Application.

[130] Chen H., Gao P., Zhu X., Basyal S., Ma L.Q. (2024). Monitoring, fate and transport, and risk assessment of organic pollutants in the environment: CREST publications during 2019–2023. Crit. Rev. Environ. Sci. Technol..

[131] Shen L., Xia B., Dai X. (2013). Residues of persistent organic pollutants in frequently-consumed vegetables and assessment of human health risk based on consumption of vegetables in Huizhou, South China. Chemosphere.

[132] Ma Z., Guan Z., Liu Q., Hu Y., Liu L., Wang B., Huang L., Li H., Yang Y., Han M., Sparks D.L. (2023). Chapter Four—Obstacles in continuous cropping: Mechanisms and control measures. Advances in Agronomy.

[133] Wang K., Lu Q., Dou Z., Chi Z., Cui D., Ma J., Wang G., Kuang J., Wang N., Zuo Y. (2024). A review of research progress on continuous cropping obstacles. Front. Agric. Sci. Eng..

[134] Huang L.-F., Song L.-X., Xia X.-J., Mao W.-H., Shi K., Zhou Y.-H., Yu J.-Q. (2013). Plant-Soil Feedbacks and Soil Sickness: From Mechanisms to Application in Agriculture. J. Chem. Ecol..

[135] Cesarano G., Zotti M., Antignani V., Marra R., Scala F., Bonanomi G. (2017). Soil sickness and negative plant-soil feedback: A reappraisal of hypotheses. J. Plant Pathol..

[136] Zhao H.-T., Li T.-P., Zhang Y., Hu J., Bai Y.-C., Shan Y.-H., Ke F. (2017). Effects of vermicompost amendment as a basal fertilizer on soil properties and cucumber yield and quality under continuous cropping conditions in a greenhouse. J. Soils Sediments.

[137] Cheng F., Ali M., Liu C., Deng R., Cheng Z. (2020). Garlic Allelochemical Diallyl Disulfide Alleviates Autotoxicity in the Root Exudates Caused by Long-Term Continuous Cropping of Tomato. J. Agric. Food Chem..

[138] Hao Z., Wang Q., Christie P., Li X. (2006). Autotoxicity potential of soils cropped continuously with watermelon. Allelopath. J..

[139] Yu J.Q. (2001). Autotoxic Potential of Cucurbit Crops. J. Crop Prod..

[140] Zhang Z., Zhang Z., Han X., Wu J., Zhang L., Wang J., Wang-Pruski G. (2020). Specific response mechanism to autotoxicity in melon (*Cucumis melo* L.) root revealed by physiological analyses combined with transcriptome profiling. Ecotoxicol. Environ. Saf..

[141] Ye S.F., Yu J.Q., Peng Y.H., Zheng J.H., Zou L.Y. (2004). Incidence of Fusarium wilt in *Cucumis sativus* L. is promoted by cinnamic acid, an autotoxin in root exudates. Plant Soil.

[142] Chen S., Zhou B., Lin S., Li X., Ye X. (2011). Accumulation of cinnamic acid and vanillin in eggplant root exudates and the relationship with continuous cropping obstacle. Afr. J. Biotechnol..

[143] Li X.-G., Ding C.-F., Hua K., Zhang T.-L., Zhang Y.-N., Zhao L., Yang Y.-R., Liu J.-G., Wang X.-X. (2014). Soil sickness of peanuts is attributable to modifications in soil microbes induced by peanut root exudates rather than to direct allelopathy. Soil Biol. Biochem..

[144] Frene J.P., Pandey B.K., Castrillo G. (2024). Under pressure: Elucidating soil compaction and its effect on soil functions. Plant Soil.

[145] Jia J., Zhang J., Li Y., Xie M., Wang G., Zhang J. (2022). Land use intensity constrains the positive relationship between soil microbial diversity and multifunctionality. Plant Soil.

[146] Hartmann M., Six J. (2023). Soil structure and microbiome functions in agroecosystems. Nat. Rev. Earth Environ..

[147] Shen W., Hu M., Qian D., Xue H., Gao N., Lin X. (2021). Microbial deterioration and restoration in greenhouse-based intensive vegetable production systems. Plant Soil.

[148] Fierer N., Bradford M.A., Jackson R.B. (2007). Toward an ecological classification of soil bacteria. Ecology.

[149] Griffiths B.S., Philippot L. (2013). Insights into the resistance and resilience of the soil microbial community. FEMS Microbiol. Rev..

[150] Ferris H., Tuomisto H. (2015). Unearthing the role of biological diversity in soil health. Soil Biol. Biochem..

[151] Ranjard L., Poly F., Combrisson J., Richaume A., Gourbiere F., Thioulouse J., Nazaret S. (2000). Heterogeneous cell density and genetic structure of bacterial pools associated with various soil microenvironments as determined by enumeration and DNA fingerprinting approach (RISA). Microb. Ecol..

[152] Longepierre M., Feola Conz R., Barthel M., Bru D., Philippot L., Six J., Hartmann M. (2022). Mixed Effects of Soil Compaction on the Nitrogen Cycle Under Pea and Wheat. Front. Microbiol..

[153] Hartmann M., Niklaus P.A., Zimmermann S., Schmutz S., Kremer J., Abarenkov K., Lüscher P., Widmer F., Frey B. (2014). Resistance and resilience of the forest soil microbiome to logging-associated compaction. ISME J..

[154] Hartmann M., Howes C.G., VanInsberghe D., Yu H., Bachar D., Christen R., Henrik Nilsson R., Hallam S.J., Mohn W.W. (2012). Significant and persistent impact of timber harvesting on soil microbial communities in Northern coniferous forests. ISME J..

[155] Smith S.E., Read D.J. (2008). Mycorrhizal Symbiosis.

[156] Bonfante P., Genre A. (2010). Mechanisms underlying beneficial plant–fungus interactions in mycorrhizal symbiosis. Nat. Commun..

[157] Drewry J., Cameron K., Buchan G. (2008). Pasture yield and soil physical property responses to soil compaction from treading and grazing—A review. Soil Res..

[158] Song Y., Xu M., Li X., Bian Y., Wang F., Yang X., Gu C., Jiang X. (2018). Long-Term Plastic Greenhouse Cultivation Changes Soil Microbial Community Structures: A Case Study. J. Agric. Food Chem..

[159] Owojori O.J., Reinecke A.J., Voua-Otomo P., Reinecke S.A. (2009). Comparative study of the effects of salinity on life-cycle parameters of four soil-dwelling species (*Folsomia candida*, *Enchytraeus doerjesi*, *Eisenia fetida* and *Aporrectodea caliginosa*). Pedobiologia.

[160] Liu Y., He B., Xiao Q., Wang X., Lin X., Hu J. (2023). Earthworms facilitated pepper (*Capsicum annuum* L.) growth via enhancing the population and function of arbuscular mycorrhizal fungi in a low-density polyethylene-contaminated soil. Chem. Biol. Technol. Agric..

[161] Cheng Y., Ishimoto K., Kuriyama Y., Osaki M., Ezawa T. (2013). Ninety-year-, but not single, application of phosphorus fertilizer has a major impact on arbuscular mycorrhizal fungal communities. Plant Soil.

[162] Shen W.-S., Lin X.-G. (2011). Differences in microbial community in cucumber rhizosphere soil between three fields under different land use. Acta Pedol. Sin..

[163] Shahid M., Khan M.S. (2022). Ecotoxicological implications of residual pesticides to beneficial soil bacteria: A review. Pestic. Biochem. Physiol..

[164] Shen W., Ni Y., Gao N., Bian B., Zheng S., Lin X., Chu H. (2016). Bacterial community composition is shaped by soil secondary salinization and acidification brought on by high nitrogen fertilization rates. Appl. Soil Ecol..

[165] Hu J.-L., Lin X.-G., Wang J.-H., Shen W.-S., Wu S., Peng S.-P., Mao T.-T. (2010). Arbuscular Mycorrhizal Fungal Inoculation Enhances Suppression of Cucumber Fusarium Wilt in Greenhouse Soils. Pedosphere.

[166] Shen W., Lin X., Gao N., Zhang H., Yin R., Shi W., Duan Z. (2008). Land use intensification affects soil microbial populations, functional diversity and related suppressiveness of cucumber Fusarium wilt in China’s Yangtze River Delta. Plant Soil.

[167] Sah S., Krishnani S., Singh R. (2021). Pseudomonas mediated nutritional and growth promotional activities for sustainable food security. Curr. Res. Microb. Sci..

[168] Miljaković D., Marinković J., Balešević-Tubić S. (2020). The Significance of *Bacillus* spp. in Disease Suppression and Growth Promotion of Field and Vegetable Crops. Microorganisms.

[169] Saxena A.K., Kumar M., Chakdar H., Anuroopa N., Bagyaraj D.J. (2020). *Bacillus* species in soil as a natural resource for plant health and nutrition. J. Appl. Microbiol..

[170] Zhang Y., Ye C., Su Y., Peng W., Lu R., Liu Y., Huang H., He X., Yang M., Zhu S. (2022). Soil Acidification caused by excessive application of nitrogen fertilizer aggravates soil-borne diseases: Evidence from literature review and field trials. Agric. Ecosyst. Environ..

[171] Li S., Liu Y., Wang J., Yang L., Zhang S., Xu C., Ding W. (2017). Soil Acidification Aggravates the Occurrence of Bacterial Wilt in South China. Front. Microbiol..

[172] Giller K.E., Witter E., McGrath S.P. (2009). Heavy metals and soil microbes. Soil Biol. Biochem..

[173] Jacobsen C.S., Hjelmsø M.H. (2014). Agricultural soils, pesticides and microbial diversity. Curr. Opin. Biotechnol..

[174] Yang Y., Song Y., Scheller H.V., Ghosh A., Ban Y., Chen H., Tang M. (2015). Community structure of arbuscular mycorrhizal fungi associated with *Robinia pseudoacacia* in uncontaminated and heavy metal contaminated soils. Soil Biol. Biochem..

[175] Leyval C., Turnau K., Haselwandter K. (1997). Effect of heavy metal pollution on mycorrhizal colonization and function: Physiological, ecological and applied aspects. Mycorrhiza.

[176] Beaumelle L., Tison L., Eisenhauer N., Hines J., Malladi S., Pelosi C., Thouvenot L., Phillips H.R.P. (2023). Pesticide effects on soil fauna communities—A meta-analysis. J. Appl. Ecol..

[177] Yang T., Lupwayi N., Marc S.-A., Siddique K.H.M., Bainard L.D. (2021). Anthropogenic drivers of soil microbial communities and impacts on soil biological functions in agroecosystems. Glob. Ecol. Conserv..

[178] Pang S., Lin Z., Zhang W., Mishra S., Bhatt P., Chen S. (2020). Insights into the Microbial Degradation and Biochemical Mechanisms of Neonicotinoids. Front. Microbiol..

[179] Mata L., Knapp R.A., McDougall R., Overton K., Hoffmann A.A., Umina P.A. (2024). Acute toxicity effects of pesticides on beneficial organisms—Dispelling myths for a more sustainable use of chemicals in agricultural environments. Sci. Total Environ..

[180] Wang G., Li X., Xi X., Cong W.-F. (2022). Crop diversification reinforces soil microbiome functions and soil health. Plant Soil.

[181] Gu X., Yang N., Zhao Y., Liu W., Li T. (2022). Long-term watermelon continuous cropping leads to drastic shifts in soil bacterial and fungal community composition across gravel mulch fields. BMC Microbiol..

[182] Shen T., Zhang X., Li L., Qi Y., Ji H., Yang G., Zhang X.-X. (2024). Dynamic Changes in Rhizosphere Microbial Communities of Watermelon during Continuous Monocropping with Gravel Mulch. J. Soil Sci. Plant Nutr..

[183] Wei Z., Yang T., Friman V.-P., Xu Y., Shen Q., Jousset A. (2015). Trophic network architecture of root-associated bacterial communities determines pathogen invasion and plant health. Nat. Commun..

[184] Chen P., Wang Y.-Z., Liu Q.-Z., Zhang Y.-T., Li X.-Y., Li H.-Q., Li W.-H. (2020). Phase changes of continuous cropping obstacles in strawberry (*Fragaria × ananassa* Duch.) production. Appl. Soil Ecol..

[185] Tian X., Zhao X., Mao Z., Xie B. (2020). Variation and Dynamics of Soil Nematode Communities in Greenhouses with Different Continuous Cropping Periods. Hortic. Plant J..

[186] Ku Y., Li W., Mei X., Yang X., Cao C., Zhang H., Cao L., Li M. (2022). Biological Control of Melon Continuous Cropping Obstacles: Weakening the Negative Effects of the Vicious Cycle in Continuous Cropping Soil. Microbiol. Spectr..

[187] Desaeger J.A., Bui H.X. (2022). Root-knot nematode damage to a cucurbit double crop is increased by chloropicrin fumigation on the previous tomato crop. Pest Manag. Sci..

[188] Hou D., O’Connor D., Igalavithana A.D., Alessi D.S., Luo J., Tsang D.C.W., Sparks D.L., Yamauchi Y., Rinklebe J., Ok Y.S. (2020). Metal contamination and bioremediation of agricultural soils for food safety and sustainability. Nat. Rev. Earth Environ..

[189] Wen D., Fu R., Li Q. (2021). Removal of inorganic contaminants in soil by electrokinetic remediation technologies: A review. J. Hazard. Mater..

[190] Chen Y., Camps-Arbestain M., Shen Q., Singh B., Cayuela M.L. (2018). The long-term role of organic amendments in building soil nutrient fertility: A meta-analysis and review. Nutr. Cycl. Agroecosyst..

[191] Dahal S., Manandhar B. (2021). Soil management practices in commercial vegetable farming in changing socioeconomic context in Makawanpur, Nepal. Environ. Chall..

[192] Luan H., Gao W., Huang S., Tang J., Li M., Zhang H., Chen X., Masiliūnas D. (2020). Organic amendment increases soil respiration in a greenhouse vegetable production system through decreasing soil organic carbon recalcitrance and increasing carbon-degrading microbial activity. J. Soils Sediments.

[193] Wang X., Bian Q., Jiang Y., Zhu L., Chen Y., Liang Y., Sun B. (2021). Organic amendments drive shifts in microbial community structure and keystone taxa which increase C mineralization across aggregate size classes. Soil Biol. Biochem..

[194] Norris C.E., Congreves K.A. (2018). Alternative Management Practices Improve Soil Health Indices in Intensive Vegetable Cropping Systems: A Review. Front. Environ. Sci..

[195] Xu L.Y., Wang M.Y., Shi X.Z., Yu Q.B., Shi Y.J., Xu S.X., Sun W.X. (2018). Effect of long-term organic fertilization on the soil pore characteristics of greenhouse vegetable fields converted from rice-wheat rotation fields. Sci. Total Environ..

[196] Du Y., Cui B., Zhang Q., Wang Z., Sun J., Niu W. (2020). Effects of manure fertilizer on crop yield and soil properties in China: A meta-analysis. Catena.

[197] Thompson A., Goyne K.W. Introduction to the Sorption of Chemical Constituents in Soils. https://www.nature.com/scitable/knowledge/library/introduction-to-the-sorption-of-chemical-constituents-94841002.

[198] Shi R.-Y., Liu Z.-D., Li Y., Jiang T., Xu M., Li J.-Y., Xu R.-K. (2019). Mechanisms for increasing soil resistance to acidification by long-term manure application. Soil Tillage Res..

[199] Siedt M., Schäffer A., Smith K.E.C., Nabel M., Roß-Nickoll M., van Dongen J.T. (2021). Comparing straw, compost, and biochar regarding their suitability as agricultural soil amendments to affect soil structure, nutrient leaching, microbial communities, and the fate of pesticides. Sci. Total Environ..

[200] FAO (2022). Soils for Nutrition: State of the Art.

[201] Warman P.R. (2005). Soil Fertility, Yield and Nutrient Contents of Vegetable Crops after 12 Years of Compost or Fertilizer Amendments. Biol. Agric. Hortic..

[202] Zhang Y.-J., Gao W., Luan H.-A., Tang J.-W., Li R.-N., Li M.-Y., Zhang H.-Z., Huang S.-W. (2022). Effects of a decade of organic fertilizer substitution on vegetable yield and soil phosphorus pools, phosphatase activities, and the microbial community in a greenhouse vegetable production system. J. Integr. Agric..

[203] Diacono M., Montemurro F. (2010). Long-term effects of organic amendments on soil fertility. A review. Agron. Sustain. Dev..

[204] Luan H., Gao W., Huang S., Tang J., Li M., Zhang H., Chen X. (2019). Partial substitution of chemical fertilizer with organic amendments affects soil organic carbon composition and stability in a greenhouse vegetable production system. Soil Tillage Res..

[205] Wang S., Hu K., Feng P., Qin W., Leghari S.J. (2023). Determining the effects of organic manure substitution on soil pH in Chinese vegetable fields: A meta-analysis. J. Soils Sediments.

[206] Amlinger F., Götz B., Dreher P., Geszti J., Weissteiner C. (2003). Nitrogen in biowaste and yard waste compost: Dynamics of mobilisation and availability—A review. Eur. J. Soil Biol..

[207] Seufert V., Ramankutty N., Foley J.A. (2012). Comparing the yields of organic and conventional agriculture. Nature.

[208] Chen X., Cui Z., Fan M., Vitousek P., Zhao M., Ma W., Wang Z., Zhang W., Yan X., Yang J. (2014). Producing more grain with lower environmental costs. Nature.

[209] Wang X., Dou Z., Shi X., Zou C., Liu D., Wang Z., Guan X., Sun Y., Wu G., Zhang B. (2021). Innovative management programme reduces environmental impacts in Chinese vegetable production. Nat. Food.

[210] Kutman U.B., Rengel Z., Cakmak I., White P.J. (2023). Chapter 9—Mineral nutrition and crop quality. Marschner’s Mineral Nutrition of Plants.

[211] Lehmann J., Kleber M. (2015). The contentious nature of soil organic matter. Nature.

[212] Cerecetto V., Smalla K., Nesme J., Garaycochea S., Fresia P., Sørensen S.J., Babin D., Leoni C. (2021). Reduced tillage, cover crops and organic amendments affect soil microbiota and improve soil health in Uruguayan vegetable farming systems. FEMS Microbiol. Ecol..

[213] Li X., Zhu W., Xu F., Du J., Tian X., Shi J., Wei G. (2021). Organic amendments affect soil organic carbon sequestration and fractions in fields with long-term contrasting nitrogen applications. Agric. Ecosyst. Environ..

[214] Lucas S.T., D’Angelo E.M., Williams M.A. (2014). Improving soil structure by promoting fungal abundance with organic soil amendments. Appl. Soil Ecol..

[215] Neher D.A., Weicht T.R., Dunseith P. (2014). Compost for Management of Weed Seeds, Pathogen, and Early Blight on Brassicas in Organic Farmer Fields. Agroecol. Sustain. Food Syst..

[216] Mamphogoro T.P., Babalola O.O., Aiyegoro O.A. (2020). Sustainable management strategies for bacterial wilt of sweet peppers (*Capsicum annuum*) and other Solanaceous crops. J. Appl. Microbiol..

[217] Ma X., Du M., Liu P., Tang Y., Li H., Yuan Q., Ruan Y., Meng L., Zhang J., Lin M. (2021). Alternation of soil bacterial and fungal communities by tomato–rice rotation in Hainan Island in Southeast of China. Arch. Microbiol..

[218] Ntalli N., Caboni P. (2017). A review of isothiocyanates biofumigation activity on plant parasitic nematodes. Phytochem. Rev..

[219] Sennett L., Burton D.L., Goyer C., Zebarth B.J. (2021). Influence of chemical fumigation and biofumigation on soil nitrogen cycling processes and nitrifier and denitrifier abundance. Soil Biol. Biochem..

[220] Gamliel A., van Bruggen A.H.C. (2016). Maintaining soil health for crop production in organic greenhouses. Sci. Hortic..

[221] Wang Q., Ma Y., Yang H., Chang Z. (2014). Effect of biofumigation and chemical fumigation on soil microbial community structure and control of pepper Phytophthora blight. World J. Microbiol. Biotechnol..

[222] Liu X., Ren X., Tang S., Zhang Z., Huang Y., Sun Y., Gao Z., Ma Z. (2023). Effects of Broccoli Rotation on Soil Microbial Community Structure and Physicochemical Properties in Continuous Melon Cropping. Agronomy.

[223] Ghani M.I., Ali A., Atif M.J., Pathan S.I., Pietramellara G., Ali M., Amin B., Cheng Z. (2022). Diversified crop rotation improves continuous monocropping eggplant production by altering the soil microbial community and biochemical properties. Plant Soil.

[224] Munkholm L.J., Heck R.J., Deen B. (2013). Long-term rotation and tillage effects on soil structure and crop yield. Soil Tillage Res..

[225] Yan Y., Cheng-Hua L., Zhong-Jian P. (2015). Effect of greenhouse soil management on soil aggregation and organic matter in northeast China. Catena.

[226] Bindraban P.S., Dimkpa C., Nagarajan L., Roy A., Rabbinge R. (2015). Revisiting fertilisers and fertilisation strategies for improved nutrient uptake by plants. Biol. Fertil. Soils.

[227] Rusu O.-R., Mangalagiu I., Amăriucăi-Mantu D., Teliban G.-C., Cojocaru A., Burducea M., Mihalache G., Roșca M., Caruso G., Sekara A. (2023). Interaction Effects of Cultivars and Nutrition on Quality and Yield of Tomato. Horticulturae.

[228] Whipps J.M. (2001). Microbial interactions and biocontrol in the rhizosphere. J. Exp. Bot..

[229] Shen H., Zhang C.-L., Zhang Y.-Y. (2019). Research advance of paddy-upland rotation for greenhouse vegetables. Mod. Agric. Sci. Technol..

[230] Zhou N., Chen Y., Wang J., Yang W., Wang Y. (2023). Reducing Chemical Fertilizer Application in Greenhouse Vegetable Cultivation under Different Residual Levels of Nutrient. Agriculture.

[231] Lakshani N., Wijerathne H., Sandaruwan C., Kottegoda N., Karunarathne V. (2023). Release Kinetic Models and Release Mechanisms of Controlled-Release and Slow-Release Fertilizers. ACS Agric. Sci. Technol..

[232] Niu J., Liu C., Huang M., Liu K., Yan D. (2021). Effects of Foliar Fertilization: A Review of Current Status and Future Perspectives. J. Soil Sci. Plant Nutr..

[233] An Z., Yang Y., Yang X., Ma W., Jiang W., Li Y., Chen G., Zhang W., Zhuang M., Wang C. (2024). Promoting sustainable smallholder farming via multistakeholder collaboration. Proc. Natl. Acad. Sci. USA.

